# Suppressing miR-21 activity in tumor-associated macrophages promotes an antitumor immune response

**DOI:** 10.1172/JCI127125

**Published:** 2019-11-11

**Authors:** Mahnaz Sahraei, Balkrishna Chaube, Yuting Liu, Jonathan Sun, Alanna Kaplan, Nathan L. Price, Wen Ding, Stanley Oyaghire, Rolando García-Milian, Sameet Mehta, Yana K. Reshetnyak, Raman Bahal, Paolo Fiorina, Peter M. Glazer, David L. Rimm, Carlos Fernández-Hernando, Yajaira Suárez

**Affiliations:** 1 Department of Comparative Medicine,; 2 Program in Integrative Cell Signaling and Neurobiology of Metabolism (ICSNM),; 3 Vascular Biology and Therapeutics Program (VBT),; 4 Department of Pathology,; 5 Department of Therapeutic Radiology,; 6 Bioinformatics Support Program, Cushing/Whitney Medical Library, and; 7 Yale Center for Genome Analysis, Yale University School of Medicine, New Haven, Connecticut, USA.; 8 Physics Department, University of Rhode Island, Kingston, Rhode Island, USA.; 9 Department of Pharmaceutical Sciences, University of Connecticut, Storrs, Connecticut, USA.; 10 Division of Nephrology, Boston Children’s Hospital, Harvard Medical School, Boston, Massachusetts, USA.

**Keywords:** Angiogenesis, Immunology, Cancer, Macrophages

## Abstract

microRNA-21 (miR-21) is the most commonly upregulated miRNA in solid tumors. This cancer-associated microRNA (oncomiR) regulates various downstream effectors associated with tumor pathogenesis during all stages of carcinogenesis. In this study, we analyzed the function of miR-21 in noncancer cells of the tumor microenvironment to further evaluate its contribution to tumor progression. We report that the expression of miR-21 in cells of the tumor immune infiltrate, and in particular in macrophages, was responsible for promoting tumor growth. Absence of miR-21 expression in tumor- associated macrophages (TAMs), caused a global rewiring of their transcriptional regulatory network that was skewed toward a proinflammatory angiostatic phenotype. This promoted an antitumoral immune response characterized by a macrophage-mediated improvement of cytotoxic T-cell responses through the induction of cytokines and chemokines, including IL-12 and C-X-C motif chemokine 10. These effects translated to a reduction in tumor neovascularization and an induction of tumor cell death that led to decreased tumor growth. Additionally, using the carrier peptide pH (low) insertion peptide, we were able to target miR-21 in TAMs, which decreased tumor growth even under conditions where miR-21 expression was deficient in cancer cells. Consequently, miR-21 inhibition in TAMs induced an angiostatic and immunostimulatory activation with potential therapeutic implications.

## Graphical abstract

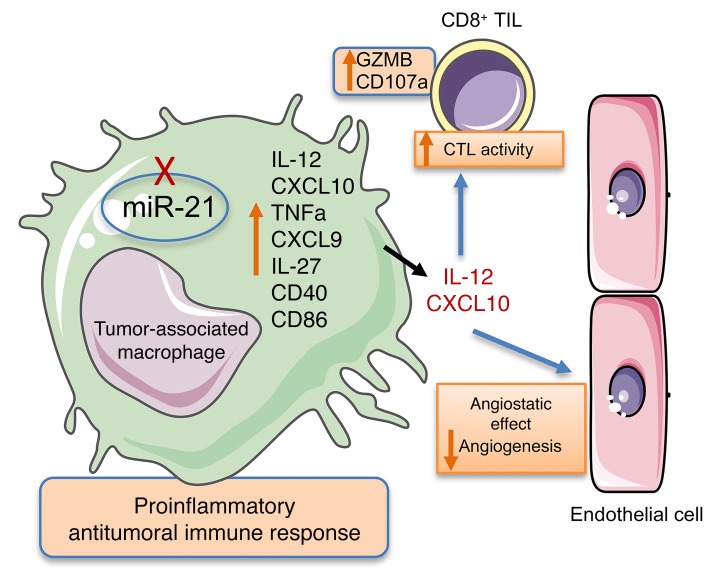

## Introduction

miRNAs are small endogenous non–protein-coding RNAs that posttranscriptionally regulate the expression of multiple target genes in different cell types. In this manner, miRNAs control many physiological cellular processes, as well as several pathologies. Widespread dysregulation of miRNA expression has been reported in tumor cells, with either an oncogenic or a tumor suppressive role (1). Given their pivotal role in tumor initiation, progression, and response to therapy, these molecules have been accepted as potential cancer biomarkers (2). During neoplastic development, miRNAs have also been reported to be dysregulated in cells from the tumor microenvironment (TME) (3). Therefore, their role in cancer progression is not just limited to regulating cancer cell behavior. The nonmalignant cells of the TME have a dynamic and often tumor-supporting function (4), thus contributing to the clinicopathological profile and to the efficacy of anticancer therapy. The TME contains diverse types of cells, including cells of the immune system, endothelial cells, and fibroblasts. Recent studies have begun to unravel the cell-autonomous significance of miRNAs in macrophages, T cells, and endothelial cells (ECs) and their potential implications for cancer (5–7).

miR-21 is the most commonly upregulated miRNA in solid tumors and hematological malignancies, and many studies have linked this cancer-associated microRNA (oncomiR) to poor prognosis and survival (8). The contribution of miR-21 to tumorigenesis has been extensively studied in the context of cancer cells, as it regulates various downstream effectors associated with tumor pathogenesis including cell invasion, proliferation, migration, apoptosis, and chemoresistance (8, 9). The role of miR-21 in cells of the TME, however, has not been comprehensively studied. miR-21 is the most abundant miRNA in macrophages (10) and highly expressed in ECs and T lymphocytes (11, 12). Several reports have described the role of miR-21 in regulating cellular functions that may have an impact on tumor growth, such as the inflammatory responses of macrophages, T-cell activation and endothelial angiogenic functions (12–18). However, the conclusions of these reports are inconsistent and in most cases are not directly studied in the context of tumor progression. In other instances, the studies relied on culture systems to inhibit or overexpress miR-21, or approaches to globally target miR-21 in a non–cell/tissue-specific manner (19–23). Recent attempts to understand the role of miR-21 within immune infiltrating cells have also yielded conflicting results mostly due to lack of experimental data on cell-type specific KO or cell-type specific targeting approaches (24, 25).

In this work, we have analyzed the function of miR-21 in noncancerous cells of the TME to determine its contribution to tumor progression. To this end, we used a syngeneic tumor mouse model and comprehensively assessed the effect of miR-21 deficiency in different cells/tissues on tumor progression. Briefly, our results indicate that the expression of miR-21 in macrophages of the tumor immune infiltrate plays a pivotal role in the regulation of tumor progression. Specifically, we found that both deletion and targeted inhibition of miR-21 in tumor-associated macrophages promotes proinflammatory angiostatic functions. This results in reduced tumor neovascularization and an antitumoral immune response, which is characterized by a macrophage-mediated improvement of cytotoxic T-cell responses. Together, these effects led to diminished tumor growth.

## Results

### miR-21–deficient mice develop smaller tumors.

**Figure 1:**
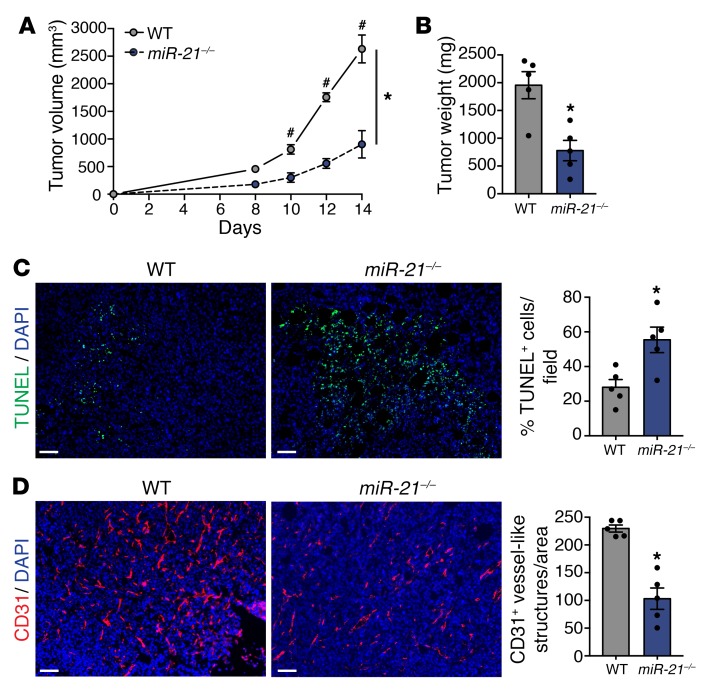
miR-21–deficient mice develop smaller tumors. (**A**–**D**) Tumor analysis of *WT* and *miR-21^–/–^* mice with s.c. injection of LLCs in the dorsal flank (*n* = 5). Tumor volume (**A**), final tumor weight (**B**). (**C**) Representative images of TUNEL and DAPI staining of cross sections of LLC tumors. Right panel: Quantification of %DAPI^+^ TUNEL^+^ cells. (**D**) Representative images of CD31 and DAPI immunostaining. Right panel: Quantification of CD31^+^ vessel-like structures. Results are mean ± SEM. *P < 0.05. (**A**) Two-way ANOVA (time and genotype) with Bonferroni correction, ^#^*P* < 0.05 individual comparisons. (**B**–**D**) Mann-Whitney *U* test. (**A**–**D**) Representative experiments out of 3 with similar results. Scale bars: 70 μm.

In this study, we aimed to analyze the role of miR-21 in noncancerous cells of the TME on regulation of tumor progression. We used the Lewis lung carcinoma (LLC) model, which is a syngeneic, hence immunologically compatible model, and analyzed tumor growth in both *WT* and miR-21 KO mice (*miR-21^–/–^*). As shown in Figure 1, A and B, *miR-21^–/–^* mice developed tumors with reduced volume and weight when compared with *WT* controls. Tumors of *miR-21^–/–^* mice exhibited an increased number of TUNEL-positive cells (Figure 1C) and reduced tumor-associated vasculature, as shown by the diminished CD31^+^ vessel-like structures (Figure 1D). These results indicate that loss of miR-21 increases tumor cell death, diminishes tumor angiogenesis, and provides evidence that miR-21 expression in cells other than cancer cells has an important role in promoting tumor growth.

### Lack of miR-21 expression in immune cells is responsible for reduced tumor burden.

**Figure 2:**
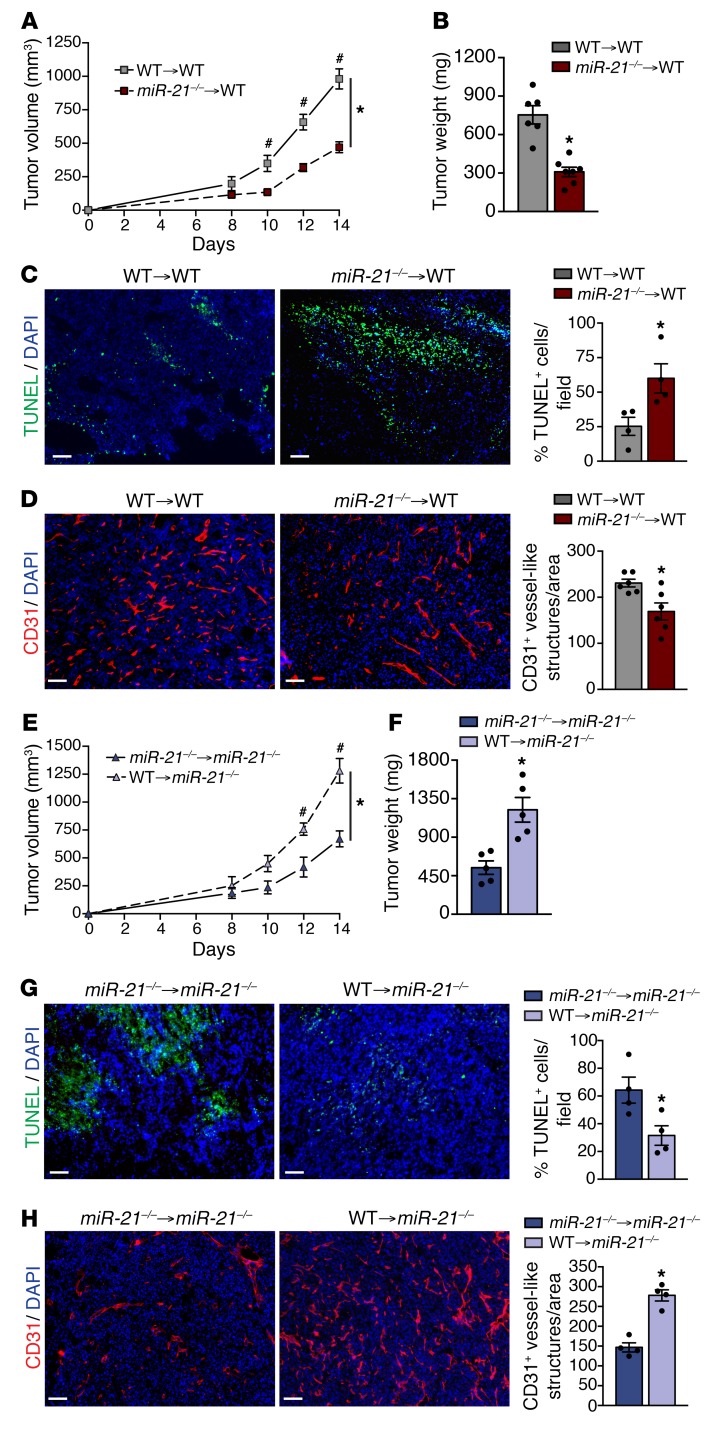
Hematopoietic miR-21 regulates and promotes tumor progression. (**A**–**D**) Tumor analysis of *WT* mice transplanted with *WT* or *miR-21^–/–^* BM and injected with LLCs s.c. (*n* = 7). LLC tumor volume (**A**), final tumor weight (**B**). (**C**) Representative images of TUNEL and DAPI staining of cross sections of LLC tumors. Right panel: Quantification of %DAPI^+^ TUNEL^+^ cells (*n* = 5 out of 7 randomly selected). (**D**) Representative images of CD31 and DAPI immunostaining. Right panel: Quantification of CD31^+^ vessel-like structures (*n* = 6 out of 7 randomly selected). (**E**–**H**) Tumor analysis of *miR-21^–/–^* mice transplanted with *WT* or *miR-21^–/–^* BM cells and injected with LLCs s.c. (*n* = 5). Tumor volume (**E**), final tumor weight (**F**). (**G**) Representative images of TUNEL and DAPI staining of cross sections of LLC tumors. Right panel: Quantification of %DAPI^+^ TUNEL^+^ cells. (**H**) Representative images of CD31 and DAPI immunostaining. Right panel: Quantification of CD31^+^ vessel-like structures (*n* = 4 out of 5 randomly selected). Results are mean ± SEM. *P < 0.05. (**A** and **E**) Two-way ANOVA (time and genotype) with Bonferroni correction, ^#^*P* < 0.05 individual comparisons. (**B**–**D** and **F**–**H**) Mann-Whitney *U* test. (**A**–**H**) Representative experiments out of 2 with similar results. Scale bars: 70 μm.

To eliminate the role of stromal cells (e.g., fibroblasts, ECs) in limiting tumor growth of *miR-21^–/–^* mice, *WT* mice were lethally irradiated and subsequently transplanted with *WT* or *miR-21^–/–^* BM. Mice transplanted with *miR-21^–/–^* BM developed smaller tumors (Figure 2, A and B). Histological analysis of their tumors revealed both increased TUNEL-positive cells and decreased vascularization (Figure 2, C and D). Interestingly, a reverse transplant of *WT* or *miR-21^–/–^* BM into *miR-21^–/–^* mice resulted in larger tumors in mice transplanted with *WT* BM (Figure 2, E and F), with decreased TUNEL-positive cells and increased CD31^+^ vessel- like structures (Figure 2, G and H). These results suggest that miR-21 expression within the tumor immune infiltrate is responsible for promoting tumor growth and that its deletion causes increased tumor cell death and decreased tumor angiogenesis.

### Tumor immune infiltrate of miR-21^–/–^ or WT mice adoptively transferred with miR-21^–/–^ BM is characterized by the presence of tumor-associated macrophages with an enhanced differentiated phenotype.

Then, we examined the tumor-infiltrating immune cells in LLC tumors of either *WT* or *miR-21^–/–^* mice as well as *WT* mice adoptively transferred with *WT* or *miR-21^–/–^* BM. We analyzed the frequency of immune cells related to tumor development, including myeloid-derived suppressor cells (MDSCs), tumor-infiltrating lymphocytes (TILs), and macrophages (4). We did not find differences in the percentage of MDSCs in the tumors of *miR-21^–/–^* mice or the proportion of monocytic or granulocytic MDSCs (Supplemental Figure 1A; supplemental material available online with this article; https://doi.org/10.1172/JCI127125DS1). We also did not detect differences in the percentage of CD4^+^ or CD8^+^ T cells in tumors from *miR-21^–/–^* mice or tumors from *WT* mice with *miR-21^–/–^* BM (Supplemental Figure 1, B and C). Furthermore, the proportion of IFNG expressing CD4^+^ or CD8^+^ T cells was similar in tumors from *WT* and *miR-21^–/–^* mice (Supplemental Figure 1D), suggesting that the percentage of activated T cells was equivalent.

**Figure 3:**
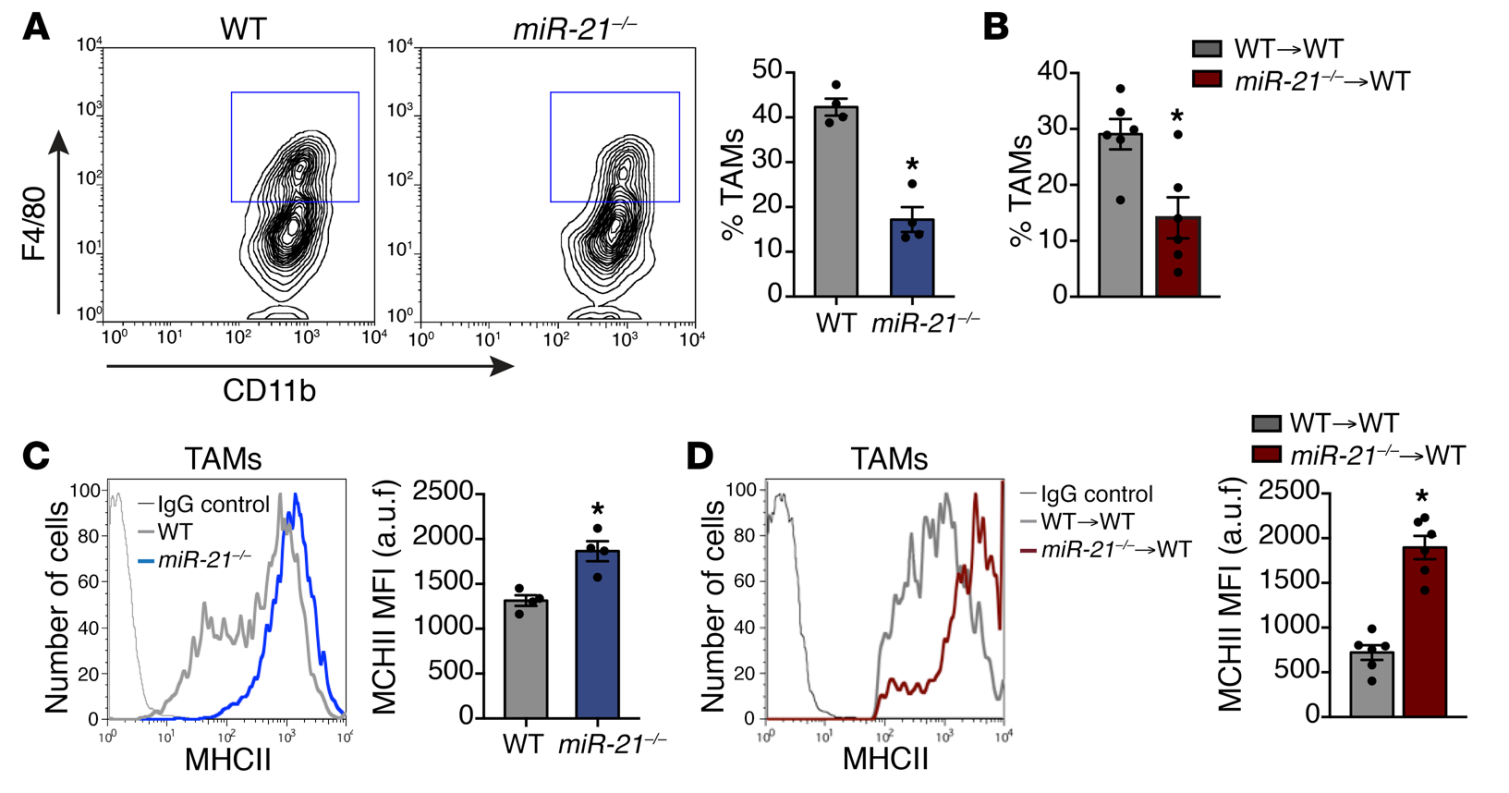
TAMs of *miR-21^–/–^* or *WT* mice adoptively transferred with *miR-21^–/–^* BM have an enhanced differentiated phenotype. (**A**) Left panel: Representative flow cytometry plots of TAM detection in s.c. LLC tumors of *WT* and *miR-21^–/–^* mice. TAMs were gated as CD45^+^ CD11b^+^ MHC II^+^ F4/80^+^ cells of the digested tumor. Right panel: Average % TAMs (*n* = 4 out of 5 randomly selected). (**B**) Average % TAMs of LLC tumors in *WT* mice transplanted with *WT* or *miR-21^–/–^* BM cells (*n* = 6 out of 7 randomly selected). (**C** and **D**) Representative histograms of MHC II surface expression in TAMs of s.c. LLC tumors of *WT* or *miR-21^–/–^* (**C**), or *WT* mice transplanted with *WT* or *miR-21^–/–^* BM cells (**D**) a.u.f., arbitrary units of fluorescence. Right panels: Average MFI (*n* = 4 out of 5 randomly selected for **C** or *n* = 6 out of 7 randomly selected for **D**). Results are mean ± SEM. (**A**–**D**) *P < 0.05, Mann-Whitney *U* test. (**A** and **D**) Representative experiments out of 3 with similar results. (**B** and **D**) Representative experiments out of 2 with similar results.

We did, however, find a significant reduction in TAM infiltration in *miR-21^–/–^* mice and mice transferred with *miR-21^–/–^* BM (Figure 3, A and B) and a decreased percentage of TAMs expressing C-C chemokine receptor type 2, although the level of surface expression was not affected (Supplemental Figure 1E). In both *miR-21^–/–^* animals and mice transferred with *miR-21^–/–^* BM, we found that infiltrated TAMs exhibited increased surface expression of MHC class II (MHC II) (Figure 3, C and D). MHC II^+high^ TAMs are better fosters of antitumor immune responses (26). Altogether, these data show that macrophages lacking miR-21 have reduced infiltration into the TME, and those present have a phenotype more associated with tumor growth suppression.

### Expression of miR-21 in TAMs regulates disease outcome.

**Figure 4:**
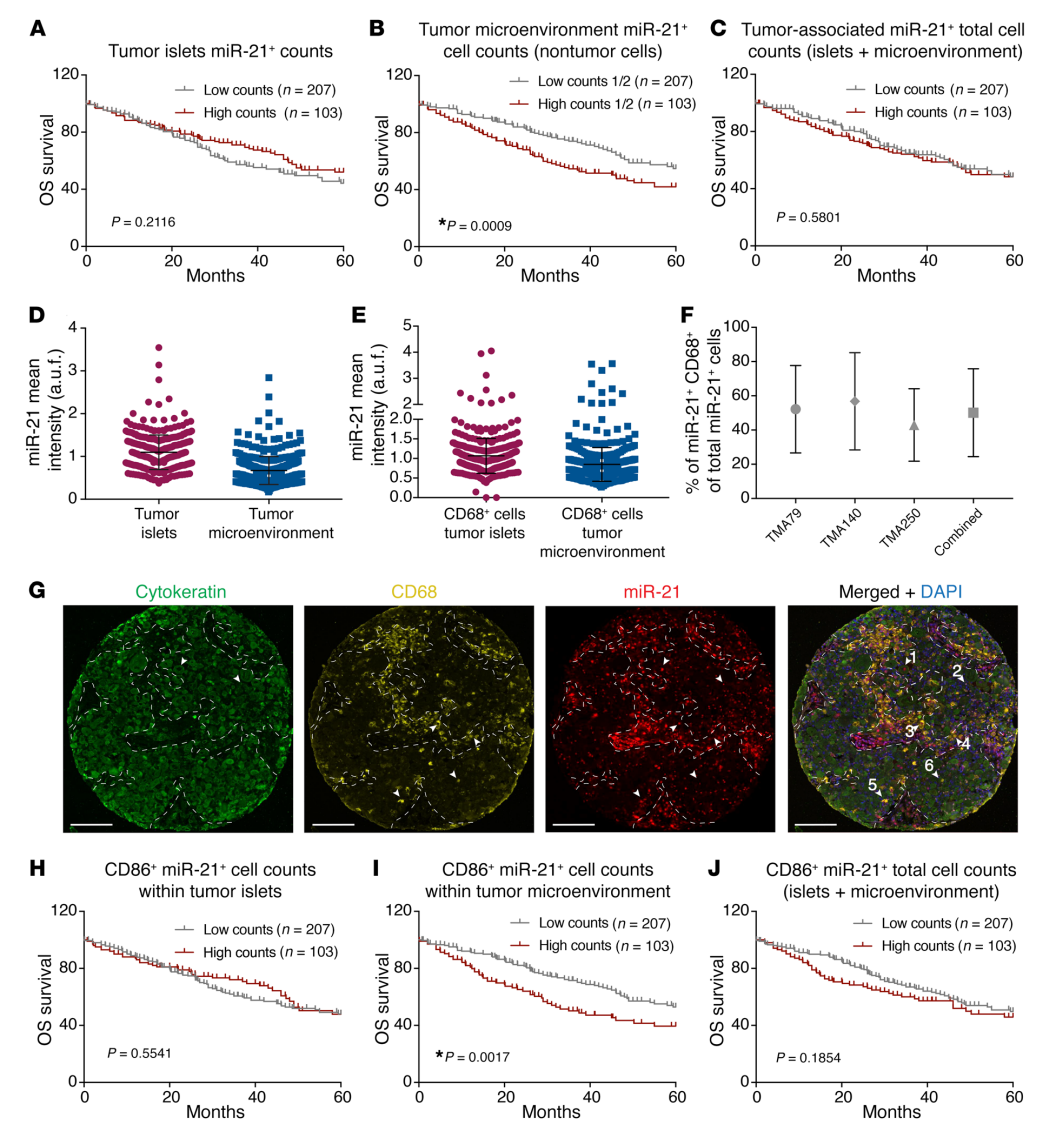
High expression of miR-21 in CD68^+^ cells of the TME of NSCLC regulates disease outcome. (**A**–**C**) Kaplan-Meier 5-year OS survival curves of patients with NSCLC in a combined analysis of 3 Yale cohorts; patients were stratified by the median of normalized miR-21–positive cell counts in (**A**) TIs, (**B**) TME, and (**C**) total cells. (**D**) Quantitative immunofluorescence of miR-21 in TI and TME cell compartments. (**E**) Quantitative immunofluorescence intensity of miR-21 in CD68^+^ cells in TI and TME cell compartments. (**F**) Analysis of the percentage of miR-21^+^ Cd68^+^ of total miR-21^+^ cells in the 3 Yale cohorts independently and combined. (**G**) Representative cytokeratin immunostaining, miR-21 FISH, and CD68 immunostaining of a random spot of YTMA250. Dashed lines delimit TI (cytokeratin-enriched areas) from TME. Arrowheads no. 1 and no. 2 indicate cytoketatin^+^ cells in TI with high or low miR-21 intensity, respectively. Arrows no. 3 and no. 4 indicate CD68^+^ cells in TME with high or low miR-21 intensity, respectively. Arrows no. 5 and no. 6 indicate CD68^+^ cells in TI with high or low miR-21 intensity, respectively. Scale bar: 100 μm. (**H**–**J**) Kaplan-Meier OS survival curves of patients with NSCLC in a combined analysis of 3 Yale cohorts; patients were stratified by the top tertile of normalized double-positive cell counts (miR-21^+^ CD68^+^) in (**H**) TI, (**I**) TME, and (**J**) total cells. (**A**–**C** and **H**–**J**) Kaplan–Meier survival curves compared by log-rank test. *P < 0.05.

miR-21 is the most consistently overexpressed miRNA across tumor types (8) and is associated with poor prognosis (27). In solid tumors, these analyses were based on total tumor RNA, which lacks critical spatial information. We used quantitative ISH (qISH) to directly assess the colocalization of miRNA expression with tumor epithelial and/or other subcellular compartments by multiplexing with DAPI and cytokeratin immunofluorescence (28, 29). To determine the prognostic value of miR-21 expression in the major tumor compartments tumor islets [TIs] vs. TME), we used 3 retrospective non–small cell lung carcinoma (NSCLC) cohorts from Yale University (Supplemental Tables 1–3). NSCLC represents the most common subtype of lung cancer (30). These tissue microarrays (TMA) termed YTMA 79, 14, or 250, are composed of surgical resection samples from 290 patients with NSCLC. In order to validate miR-21 qISH, we first assessed miR-21 qISH on heart and spleen tissue from an *miR-21^–/–^* mouse (28). No specific signal for miR-21 could be detected in heart and spleen compared with normal WT (Supplemental Figure 2A). On the TMA, we found that the expression of miR-21 in tumor epithelial cells within TI was not associated with 5-year overall survival (OS) (Figure 4A). In non-tumor cells of the TME (Figure 4B), however, there was significant prognostic effect on 5-year OS with a worse survival for patients with high levels of miR-21 expression if they were stratified by the median. Combined analysis of the expression of miR-21 in TI and the TME did not show a significant effect on OS (Figure 4C). This finding indicates that high miR-21 expression in cells of the TME (non-tumor cells) has a negative impact on OS.

We then analyzed the expression of miR-21 in both compartments and found that the level of miR-21 expression was slightly higher in TI when compared with the TME (Figure 4D). Despite this fact, high expression of miR-21 in the TME was associated with a worse prognostic value (Figure 4B). This finding is in agreement with data showing that miR-21 expression in noncancerous cells of the tumor contributes to the pathogenesis of lung cancer (31).

miR-21 is highly expressed in the monocytic/macrophage lineage (32) and the most abundant miRNA in macrophages (10). In order to determine the levels of miR-21 in immune cells of LLC tumors, we challenged *WT* mice with eGFP-LLCs and after 14 days, TAMs (CD45^+^, CD11b^+^, MHC II^+^, F4/80^+^), LLCs (eGFP^+^) and TILs (CD45^+^, F4/80^–^) were sorted and the miR-21 levels analyzed. Within the tumor, we found that miR-21 was highly expressed in TAMs compared with the rest of the immune infiltrate and tumor cells (eGFP-LLCs) (Supplemental Figure 2B). Likewise, miR-21 levels were higher in BM-derived macrophages (BMDMs) compared with LLCs in culture (Supplemental Figure 2C).

A high density of TAMs was associated with worse survival in gastric cancer and head and neck cancer but better OS in patients with colorectal cancer (33). In patients with lung cancer, conflicting data have been reported (34, 35). We used CD68 immunofluorescence to detect macrophages in our TMAs and found CD68^+^ macrophages present in all tumor TMA spots of NSCLC cohorts (*n* = 240) in both the TI and TME (Figure 4D). A total of 50.1% of all miR-21–expressing cells were CD68^+^ (Figure 4F). Although TME areas were slightly enriched in CD68^+^ miR-21^+^ cells (Figure 4G), double positive cells could be found in both compartments (Figure 4E), and the level of miR-21 expression was similar (Figure 4E). We thus analyzed the contribution of miR-21 expression in macrophages to patients’ survival. We found that the level of expression of miR-21 in CD68^+^ cells present within TI was not associated with 5-year OS (Figure 4H). When the analysis was performed within the TME (Figure 4I), however, there was significantly worse survival for patients with high levels of miR-21 in CD68^+^ cells when they were stratified by the median. Combined analysis of the expression of miR-21 in CD68^+^ cells of TI and the TME (Figure 4J) showed a slight reduction in patients’ survival when miR-21 is high in tumor macrophages. Altogether, these data indicate that although miR-21 expression in macrophages is heterogeneously localized within the tumor, the level of expression of miR-21 in macrophages and in particular, in macrophages within the TME can predict patient survival.

### miR-21–deficient TAMs express a gene signature associated with the promotion of a robust antitumor immune function.

**Figure 5:**
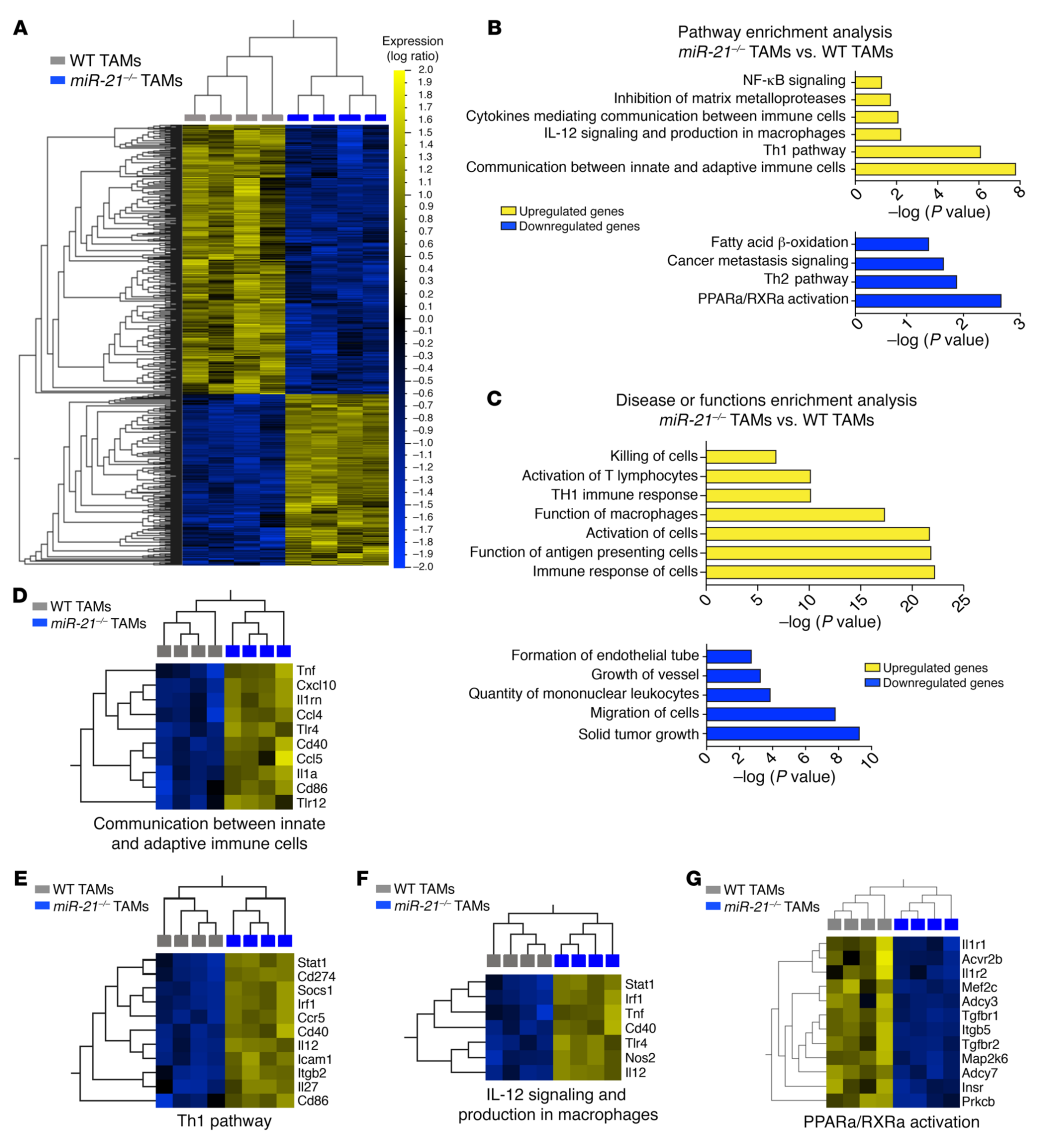
Differential gene expression of TAMs in the absence of miR-21 expression. (**A**) Heatmap of hierarchical clustering log2-transformed gene expression values of the significantly differentially expressed genes (1,035) between *miR-21^–/–^* and *WT* TAMs (*n* = 4) isolated from s.c. LLC tumors. Colors displayed by row minimum and maximum values: yellow, higher expression; blue, lower). (**B**) IPA analysis of both up- (left) and down- (right) regulated genes in LLC TAMs lacking miR-21 expression vs. *WT* TAMs. (**C**) IPA of overrepresented disease or function categories upregulated (left) and downregulated (right) as a result of miR-21 deletion in TAMs of LLC tumors. (**D**–**G**) Heatmaps of significantly differentially expressed genes that contribute to (**D**) communication between innate and adaptive immune cells (**E**), Th1 pathway (**F**), IL-12 signaling in macrophages (**G**), and PPARa/RXRa activation.

To elucidate the role of miR-21 in regulating TAM function, we isolated TAMs from LLC tumors of either *miR-21^–/–^* or *WT* mice and profiled their transcriptomes by RNA-Seq. *miR-21^–/–^* TAM gene expression patterns were distinct from *WT* TAMs (Supplemental Figure 3A). Hierarchical clustering of log ratio transformed gene expression values of 1,035 genes that were differentially and significantly expressed (FC > 1.5, *P* ≤ 0.005 B-H FDR) showed the level of transcriptional dysregulation between each genotype and highlighted the similar expression patterning found among biological replicates in each group (Figure 5A). Ingenuity pathway analysis (IPA) of both up- and downregulated genes (Figure 5B) in *miR-21^–/–^* TAMs identified gene signatures that favor cytokine-mediated communication between innate and adaptive immune cells, Th1 pathway, and a proinflammatory gene profile (Figure 5B). Conversely, a metabolic gene pattern, that in macrophages is linked to anti-inflammatory functions, and a Th2 gene signature were diminished in *miR-21^–/–^* TAMs (Figure 5B). Similarly, the top overrepresented disease and function categories pointed to an increased function of macrophages as antigen-presenting cells that promote activation of T lymphocytes (Figure 5C). Alternatively, gene signatures related to tumor growth and the regulation of blood vessel formation were downregulated (Figure 5C). Heatmaps with the list of regulated genes within these pathways and additional relevant functions are depicted in Figure 5, D–G and Supplemental Figure 3, B–D. Among the differentially expressed genes, Th1-promoting and tumor- suppressive cytokines such as *Il27* and *Il12* (36, 37) and the costimulatory molecules (38), *Cd86* and *Cd40*, were upregulated in *miR-21^–/–^* TAMs (Supplemental Figure 3E). Moreover, we found increased expression of proinflammatory mediators including *Tnf* and *Cxcl10* and *Cxcl9* (Supplemental Figure 3E).

**Figure 6:**
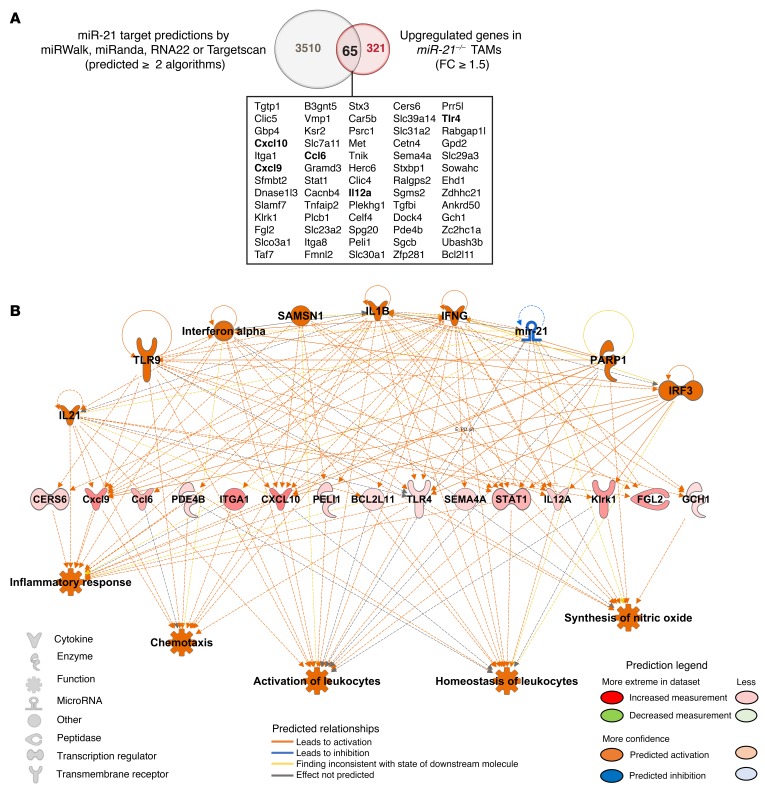
miR-21 depletion in TAMs causes a global rewiring of their transcriptional regulatory network that is skewed towards a pro-inflammatory phenotype and the promotion of an antitumoral immune response. Global correlation pattern between significantly upregulated genes in *miR-21^–/–^* TAMs and miR-21 predicted target genes. (**A**) Venn diagram depicting the correlation between miR-21 predicted targets (predicted ≥ 2 algorithms out of 4) vs. upregulated mRNAs in *miR-21^–/–^* TAMs (FC >1.5), *P* ≤ 0.005 Benjamini-Hochberg False Discovery Rate. The 65 genes of the miR-21–mRNA correlation study are depicted in the box below, including IL-12, a validated target of miR-21. (**B**) IPA network analysis of Regulator Effects algorithm of the 65 predicted targets of miR-21 significantly upregulated in *miR-21^–/–^* TAMs.

IL-12 is a reported target of mir-21 (15) and a strong inducer of Th1 responses, with demonstrated antitumor activity (37) that is produced by different immune cells including macrophages. The increased levels of *Il12* found in *miR-21^–/–^* TAMs are consistent with lost miR-21–mediated targeting. To identify miR-21/mRNA associations relevant to TAM pathophysiology, we examined global correlation patterns between significantly upregulated genes in *miR-21^–/–^* TAMs and miR-21 predicted target genes (Figure 6A). We used a combination of 4 microRNA prediction algorithms and selected genes that were predicted by at least 2 prediction methods. Among the 321 significantly upregulated mRNAs, 65 were predicted targets of miR-21 (Figure 6A). Together, they represented approximately 20% of the significantly upregulated genes found in *miR-21^–/–^* TAMs. These 65 significantly upregulated and predicted targets of miR-21 were used as a data set for network analysis using the IPA Regulator Effects algorithm (Figure 6B). Downregulation of miR-21 was predicted as an upstream regulator, providing confidence of the predicted regulatory network generated. Among the predicted upstream regulators, we found IL-21, IFNA, and IFNG, factors reported to have antitumor effects in a variety of murine experimental tumor models (39–41). The model also predicts IL-1B, a canonical proinflammatory cytokine with antitumor roles (42), as an upstream regulator. The anticipated impact of the molecular expression changes showed an increase in downstream functions involved in inflammatory responses as well as the activation and homeostasis of leukocytes. These data indicate that miR-21 depletion in TAMs causes a global rewiring of their transcriptional regulatory network that is skewed toward a proinflammatory phenotype and the promotion of an antitumoral immune response (5, 42, 43). Proinflammatory stimulation of *miR-21^–/–^* BMDMs revealed a significant upregulation of proinflammatory gene signature, including *Il12*, *Tnf*, and *Cxcl10* compared with WT BMDMs (Supplemental Figure 3F). These findings are in agreement with previous published data (10, 16) and support the idea that miR-21 deficiency primes macrophages to develop a proinflammatory program (43). In vivo, this translates to an antitumor phenotype when it happens in conjunction with Th1 immune response (5, 42). Interestingly, a proinflammatory phenotype of TAMs in patients with NSCLC has been positively associated with survival time (44).

### Increased IL12 in miR-21–deficient TAMs improves cytotoxicity of CD8^+^ T cells.

**Figure 7:**
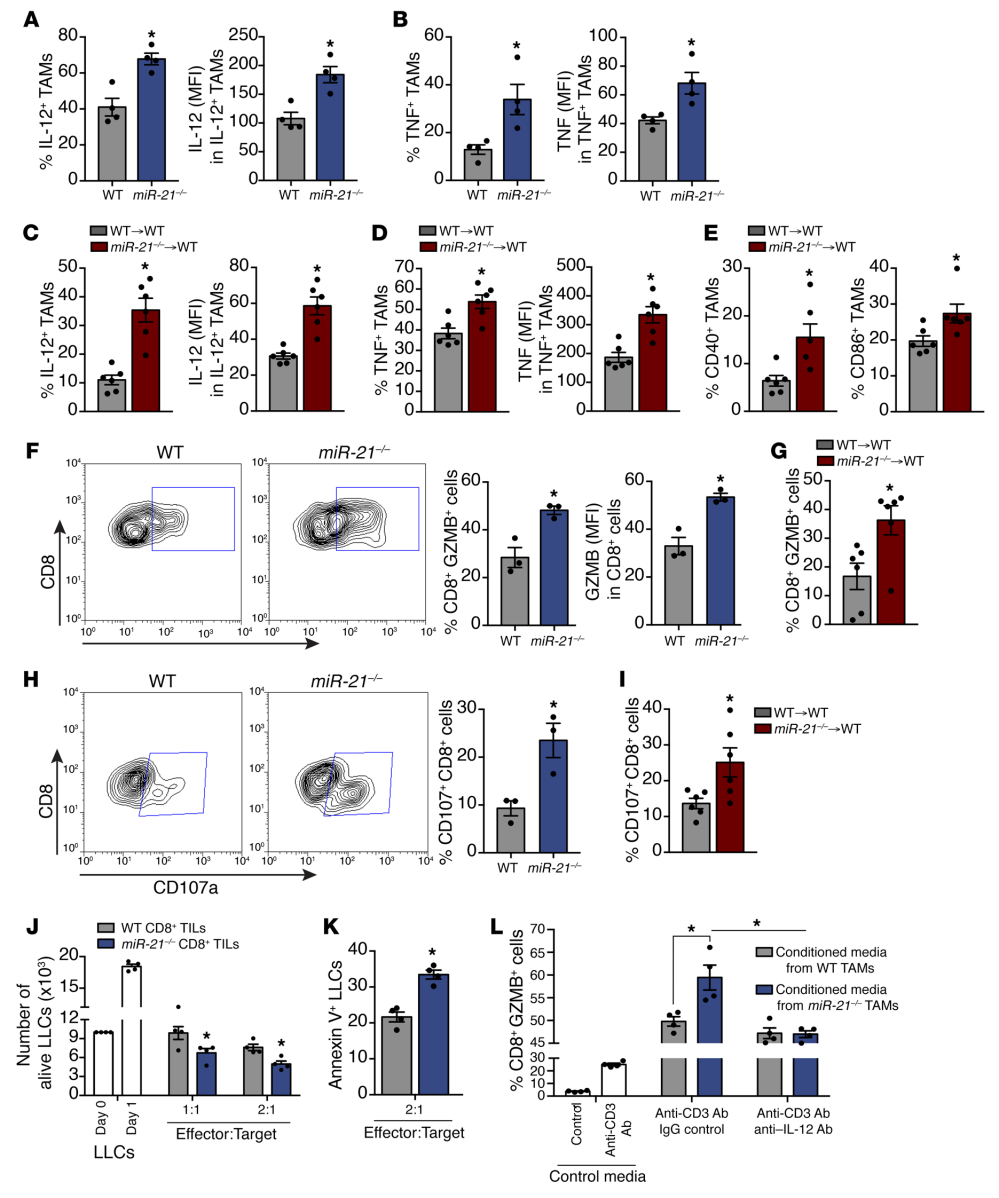
miR-21 deletion in TAMs leads to improved immune response to tumors via increased IL-12. Flow cytometry analysis (**A**) Left: % of TAMs expressing IL-12 in s.c. LLC tumors of *WT* or *miR-21^–/–^* mice. Right: IL-12 levels (MFI) (*n* = 4). (**B**) Left: % of TAMs expressing TNF. Right: TNF levels. (**C** and **D**) IL-12 and TNF (as in **A** and **B**) of s.c. LLC tumors of *WT* mice transplanted with *WT* or *miR-21^–/–^* BM, (*n* = 6). (**E**) % of TAMs expressing CD40 (left) or CD86 (right), (*n* = 6). (**F**) Left: Representative plots of GZMB in CD8^+^ TILs of s.c. LLC tumors of *WT* or *miR-21^–/–^* mice. Middle: % of CD8^+^ TILs expressing GZMB. Right: GZMB levels (*n* = 3). (**G**) % of CD8^+^ TILs expressing GZMB of s.c. LLC tumors of *WT* mice transplanted with *WT* or *miR-21^–/–^* BM (*n* = 6). (**H**) Left: Representative plots of extracellular CD107a levels in CD8^+^ TILs of s.c. LLC tumors of *WT* and *miR-21^–/–^* mice. Right: % of CD8^+^ TILs expressing extracellular CD107a (*n* = 3). (**I**) % of CD8^+^ TILs expressing extracellular CD107a in s.c. LLC tumors of *WT* mice transplanted with *WT* or *miR-21^–/–^* BM (*n* = 6). (**J**) Alive LLCs, cultured for 12 hours with CD8^+^ TILs from LLC tumors of *WT* or *miR-21^–/–^* mice at indicated effector:target ratios (*n* = 4). (**K**) % of annexin V^+^ LLCs, cultured (12 hours) with CD8^+^ TILs from LLC tumors of *WT* or *miR-21^–/–^* mice (*n* = 4). (**L**) GZMB expression by CD8^+^ splenocyte T cells activated with plate-bound anti–CD3-Ab and incubated with conditioned media from cultured *WT* or *miR-21^–/–^* TAMs plus neutralizing anti–IL-12 or IgG Ab (*n* = 4). Results are mean ± SEM. *P < 0.05. (**A**–**I** and **K**) Mann-Whitney *U* test. (**J** and **L**) Two-way ANOVA with Bonferroni correction. (**A**, **B**, **F**, and **H**). Representative experiments out of 3 or (**C**–**E**, **G**, **I**, and **J**–**L**) out of 2 with similar results.

Consistent with the TAM profiling data, we found that tumors from *miR-21^–/–^* mice had a higher percentage of IL-12^+^ and TNF^+^ TAMs (Figure 7, A and B, left panels) and also had higher levels of IL-12 and TNF (Figure 7, A and B, right panels). Similar results were observed in the tumors of *WT* mice transplanted with *miR-21^–/–^* BM (Figure 7, C and D). We also found increased levels of costimulatory molecules CD40 and CD86 (Figure 7E) linked to enhanced macrophage antitumor activity (38). These data, together with the overall transcriptome signature exhibited by *miR-21^–/–^* TAMs, suggest that these cells could participate in enhancement of cytotoxic T-lymphocyte (CTL)–dependent cell death (45–47).

Increased intracellular levels of GZMB and higher levels of extracellular LAMP1 (CD107a) in tumor-infiltrated CD8^+^ T cells indicate better targeting and killing capacity of cancer cells by CTLs (48). We found that CD8^+^ T cells within the tumors of mice transplanted with *miR-21^–/–^* BM had higher levels of GZMB than their *WT* counterparts (Figure 7, F and G) and enhanced degranulation status (Figure 7, H and I), as indicated by the larger number of CD8^+^ T cells with surface expression of CD107a (48). This finding is in agreement with the increased cell death observed in tumors of *miR-21^–/–^* or *WT* mice transplanted with *miR-21^–/–^* BM. However, the phenotype observed in CD8^+^ TILs could be mediated by the absence of miR-21 expression in these cells. Thus, we analyzed GZMB levels in CD8^+^ T cells isolated from the Peyer’s patches of healthy adult *WT* or *miR-21^–/–^* mice because in healthy and unchallenged mice, CD8^+^ T cells from Peyer’s patches express high levels of GZMB (49). We did not find differences in GZMB levels (Supplemental Figure 3G). Additionally, naïve splenic CD8^+^ T cells of *miR-21^–/–^* mice activated by anti-CD3 Ab for 3 and 6 days exhibited no difference in intracellular levels of GZMB (Supplemental Figure 3H) compared with *WT* controls. These results indicate that the absence of miR-21 in CD8^+^ T cells does not affect cell-intrinsic levels of GZMB and that additional mechanisms account for the observed differences in CD8^+^ TILs from *miR-21^–/–^* mice.

To test whether the increase of GZMB and CD107a in CD8^+^ TILs of *miR-21^–/–^* mice leads to more CTL activity, we isolated CD8^+^ TILs from *WT* or *miR-21^–/–^* tumors and immediately cultured them with LLCs to monitor their killing capacity. CD8^+^ TILs isolated from *miR-21^–/–^* mice were more effective at reducing proliferation and increasing the number of apoptotic (annexin V^+^) target cells (Figure 7, J and K), indicating that miR-21 deficiency in non-CD8^+^ cells within the TME improves CTL activity.

IL-12, a potent inducer of CTL activity in CD8^+^ T cells (37), was increased in TAMs of *miR-21^–/–^* mice or *WT* mice transplanted with *miR-21^–/–^* BM (Figure 7, A and C). Thus, we hypothesized that increased IL-12 in *miR-21^–/–^* TAMs was responsible for the increased GZMB levels. Thereby, WT splenic CD8^+^ T cells were isolated and activated with an anti-CD3 Ab and incubated with conditioned media obtained from *miR-21^–/–^* or *WT* TAMs (Figure 7L). Conditioned media from *miR-21^–/–^* TAMs led to higher levels of GZMB than media obtained from *WT* TAMs. Furthermore, a neutralizing anti–IL-12 Ab blunted this effect (Figure 7L). Altogether, these data suggest that IL-12 from *miR-21^–/–^* TAMs participates in the improved T-cell responses observed in mice transferred with *miR-21^–/–^* BM and thus contributes to the increased tumor cell death observed in these animals, consistent with the enhanced antitumor immune response mediated by *miR-21^–/–^* TAMs.

### Increased CXCL10 in miR-21–deficient TAMs mediates angiostatic effects within the LLC tumors.

**Figure 8:**
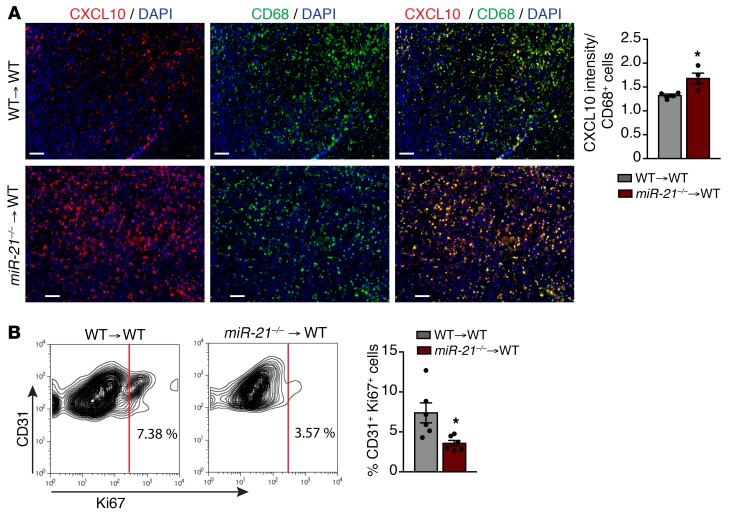
miR-21 deletion in TAMs leads decreased angiogenesis via increase of CXCL10. (**A**) Left: Representative images of immunofluorescence costaining of CD68 and CXCL10 in frozen sections from s.c. LLC tumor of *WT* mice transplanted with *WT* or *miR-21^–/–^* BM cells. Right: Quantification of CXCL10 intensity per CD68^+^ cell (*n* = 4). (**B**) left: Representative flow cytometry plot of CD45^–^CD31^+^Ki-67^+^ cells from s.c. LLC tumors of *WT* mice transplanted with *WT* or *miR-21^–/–^* BM cells. Right: Average of % CD31^+^ Ki-67^+^ cells (*n* = 6). Results are mean ± SEM. *P < 0.05. Mann-Whitney *U* test. Representative experiments out of 2 with similar results. Scale bars: 70 μm.

Reduced vascularization was another outstanding phenotype observed in tumors of mice transplanted with *miR-21^–/–^* BM. *mir-21^–/–^* TAMs exhibited a gene signature associated with decreased blood vessel formation. In addition to *Il12*, *miR-21^–/–^* TAMs exhibited increased mRNA levels of *Cxcl10* and *Cxcl9*. IL-12 in tumors negatively regulates the tumor vasculature (50). This effect is associated with IFN-inducible production of CXCL10 and CXCL9, angiostatic chemokines that have been described to halt tumor progression by inhibiting EC proliferation and differentiation into capillary structures (51). Immunofluorescent staining demonstrated that CXCL10 was significantly increased in CD68^+^ TAMs from WT mice transplanted with *miR-21^–/–^* BM (Figure 8A). Moreover, tumor ECs (TECs) isolated from these mice showed reduced numbers of Ki-67^+^ cells compared with their control counterparts (Figure 8B), indicating diminished proliferation. These results indicate that absence of miR-21 induces the expression and production of IL-12 and CXCL10 in TAMs. This can explain, at least in part, the increased tumor cell death and reduced neovascularization, leading to an overall reduction of tumor growth.

### Conditional deletion of miR-21 in macrophages reduces tumor growth by promoting CTL activity and diminishing the proliferative phenotype of tumor-associated vasculature via increased IL-12 and CXCL10 production.

To more specifically ascertain whether the observed effects were a result of deletion of miR-21 in macrophages, we used *LysMCre;miR-21^fl/fl^* mice. *LysMCre* mice allow for both fairly specific and highly efficient (83%–98%) Cre-mediated deletion of mature macrophages (52).

**Figure 9:**
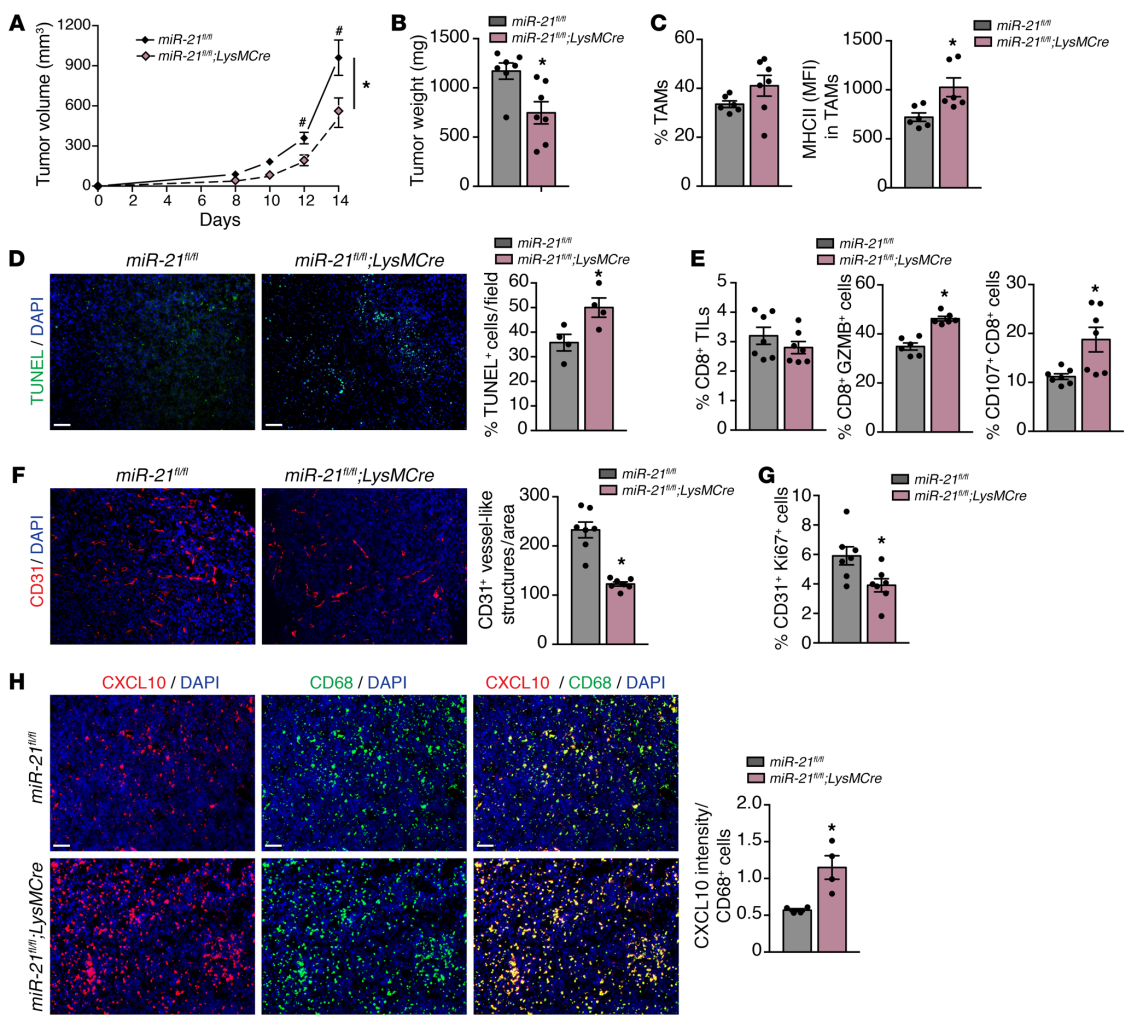
miR-21 expression by TAMs promotes tumor progression by inducing CD8^+^ T-cell suppression and angiogenesis. (**A**–**H**) Tumor analysis of *miR-21^fl/fl^* and *LysMCre;miR-21^fl/fl^* mice with s.c. injection of LLCs (*n* = 7). LLC tumor volume (**A**), final tumor weight (**B**). (**C**) Left: Average % of TAMs of s.c. LLC tumors. Right: MHC II surface levels in TAMs (average MFI) (*n* = 6 out 7 randomly selected). (**D**) Left: Representative images of TUNEL and DAPI staining of cross sections of s.c. LLC tumors. Right: Quantification of % DAPI^+^ TUNEL^+^ cells (*n* = 7). (**E**) Flow cytometry analysis of CD8^+^ TILs in s.c. LLC tumors. Average % of CD8^+^ TILs (left), % of CD8^+^ TILs expressing GZMB (middle) and % of CD8^+^ TILs with extracellular CD107a (right) (*n* = 7). (**F**) Representative images of CD31 staining of cross sections of s.c. LLC tumors. Right: Quantification of CD31^+^ vessel-like structures (*n* = 7). (**G**) Average % CD31^+^ Ki-67^+^ cells of s.c. LLC tumors (*n* = 7). (**H**) Left: Representative images of immunofluorescence costaining of CD68 and CXCL10 in frozen sections of s.c. LLC tumors. Right: Average quantification of CXCL10 intensity per CD68^+^ cell (*n* = 3, out of 7 randomly selected). Results are mean ± SEM. *P < 0.05. (**A**) Two-way ANOVA (time and genotype) with Bonferroni correction, ^#^*P* < 0.05 individual comparisons. (**B**–**H**) Mann-Whitney *U* test. (**A**–**H**) Representative experiment out of 2 with similar results. Scale bars: 70 μm.

Deletion of miR-21 in the macrophages also led to the development of smaller tumors when compared with control mice (*miR-21^fl/fl^*) (Figure 9, A and B). LLC tumors from *LysMCre;miR-21^fl/fl^* mice did not have decreased TAM infiltration; however, their MHCII expression was increased (Figure 9C). Tumors from *LysMCre;miR-21^fl/fl^* mice showed increased levels of cell death (Figure 9D), and although the overall number of CD8^+^ T cells was not affected, they had higher levels of GZMB and extracellular CD107a (Figure 9E). Tumors from *LysMCre;miR-21^fl/fl^* mice also exhibited reduced neovascularization (Figure 9F), which was associated with reduced proliferating TECs (Figure 9G) and an increased percentage of TUNEL^+^ CD31^+^ TECs(supplemental Figure 4A). This effect could also be attributable to the higher levels of CXCL10 found in CD68^+^ macrophages of these tumors (Figure 9H).

**Figure 10:**
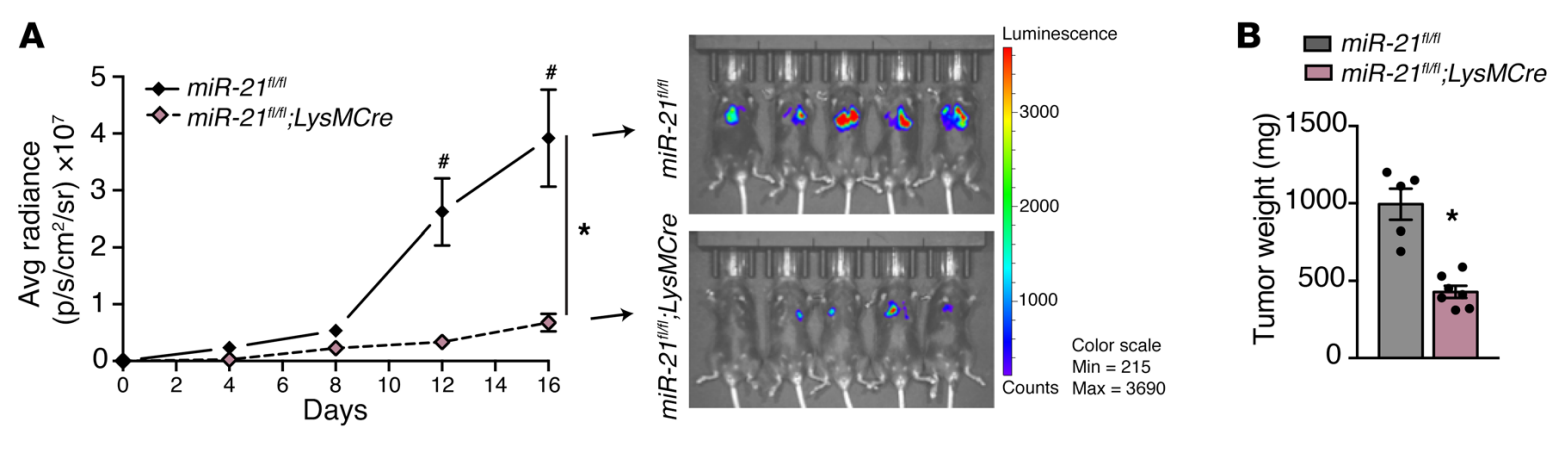
miR-21 expression by TAMs promotes tumor progression in lung tumors. (**A** and **B**) Bioluminescence imaging and final tumor weight of lung tumors generated by LL/2Red Luc injected orthotopically into the lungs of *miR-21^fl/fl^* and *LysMCre;miR-21^fl/fl^* mice. For tumor weight in **B**, remaining healthy lung tissue was removed. (*n* = 5 and 6, respectively). Results are mean ± SEM. *P < 0.05. (**A**) Two-way ANOVA (time and genotype) with Bonferroni correction, ^#^*P* < 0.05 individual comparisons. (**B**) Mann-Whitney *U* test. Representative experiment out of 2 with similar results. Scale bars: 70 μm.

To ascertain whether these effects on tumor growth were due to LLCs being grown in a non–organ-specific microenvironment, we performed orthotopic implantation of the isogenic LL/2 Red-Fluc murine LLC-derived cell line (Figure 10, A and B) or isogenic B16 melanoma murine skin cancer cell line (Supplemental Figure 4, B and C) into *LysMCre;miR-21^fl/fl^* and analyzed tumor growth. Deletion of miR-21 in macrophages led to the development of smaller tumors in these 2 different orthopic models. When cancer cells were implanted in an organ-specific microenvironment, the effect of miR-21 deficiency in macrophages was exacerbated (Figure 9, A and B, and Figure 10B).

Some reports have described LysM-mediated Cre recombination in DCs, although efficiencies range from 5% to 50% depending on the tissue and the subpopulation of DCs analyzed (53, 54). Given that tumoral DCs have been described to be necessary for CTL activation (55, 56), we analyzed LysMCre-mediated deletion of miR-21 in TAMs vs. tumor DCs. As shown in Supplemental Figure 4D, TAMs from s.c. LLC tumors of *LysMCre;miR-21^fl/fl^* showed efficient reduction of mR-21 levels when compared with TAMs isolated form control *miR-21^fl/fl^* mice. However, the expression of miR-21 in DCs isolated from *LysMCre;miR-21^fl/fl^* of *miR-21^fl/fl^* tumor-bearing mice was unaffected. Thus, the phenotype is not attributable to the deletion of miR-21 in tumor DCs.

**Figure 11:**
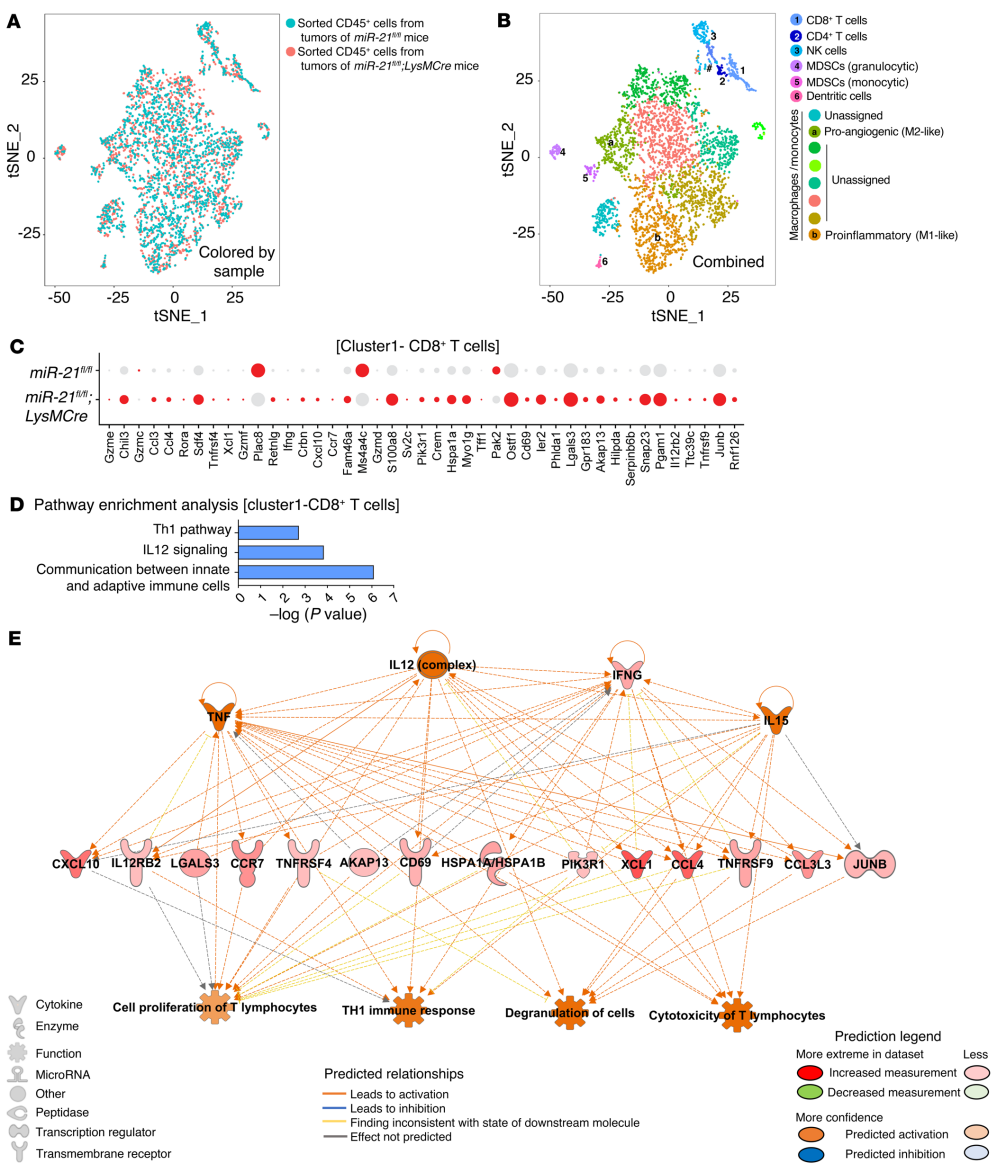
Increased cytotoxic program and phenotype in CD8^+^ TILs is the result of miR-21 deletion in macrophages. (**A**) Overlaid t-SNE plots colored by sample of CD45^+^ cells isolated from s.c. LLC tumors of *miR-21^fl/fl^* and *LysMCre;miR-21^fl/fl^* mice. (**B**) Recolored t-SNE plot based on matching results for the combined dataset distinct groups of cells separated and manifested. See feature plots of select marker gene overlays in Supplemental Figure 2A. (**C**) Dot plot analysis of differentially expressed genes in CD8^+^T cells of s.c. LLC tumor immune infiltrate of *LysMCre;miR-21^fl/fl^* or *LysMCre;miR-21^fl/fl^* mice. (**D**) IPA analysis of set of genes differentially regulated in CD8^+^ T cells from s.c. LLC tumors of *LysMCre;miR-21^fl/fl^* versus *miR-21^fl/fl^* mice. Dot size represents the fraction of cells expressing the gene, and red color represents a greater than 1.5-fold gene expression among expressing cells. (**E**) IPA network analysis using Regulator Effects algorithm of gene set differentially regulated in CD8^+^ T cells from LLC tumors of *LysMCre;miR-21^fl/fl^* versus *miR-21^fl/fl^* mice.

**Figure 12:**
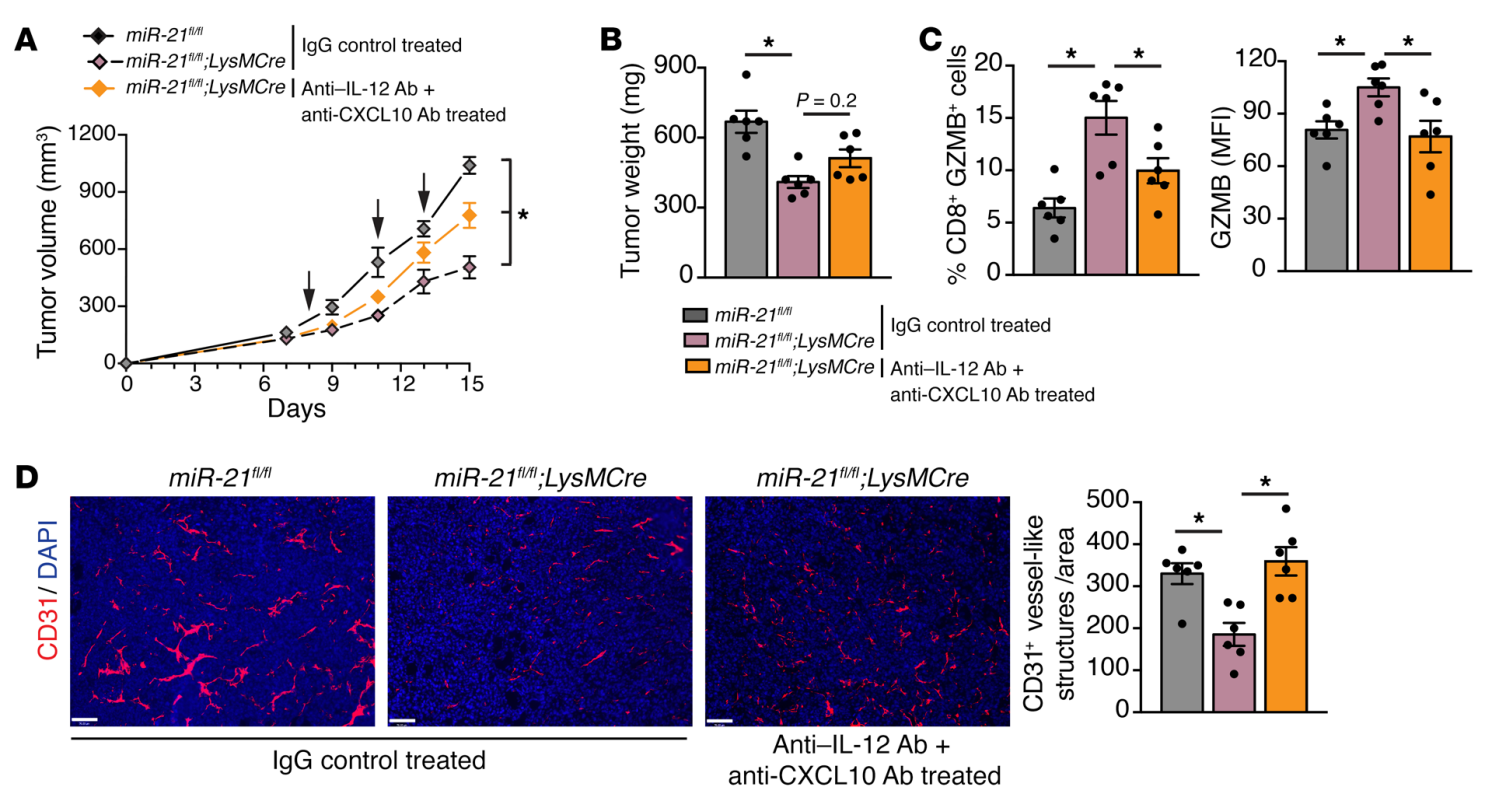
Neutralization of IL-12 and CXCL10 decreases tumor growth, improved CTL response, and decreased neovascularization in animals lacking miR-21 in macrophages. Tumor analysis of *miR-21^fl/fl^* and *LysMCre;miR-21^fl/fl^* mice with s.c. injection of LLCs (*n* = 6) and treated with 200 μg of anti–IL-12 + 100 μg of anti-CXCL10 neutralizing antibodies or 300 μg of isotype control on the indicated days. LLC tumor volume (**A**), final tumor weight (**B**). (**C**) Flow cytometry analysis of CD8^+^ TILs. Left: Average % of CD8^+^ TILs expressing GZMB, Right: GZMB levels (average MFI) (*n* = 6). (**D**) Representative images of CD31 immunostaining of cross sections. Right: Quantification of CD31^+^ vessel-like structures (*n* = 6). Results are mean ± SEM. *P < 0.05. (**A**) Two-way ANOVA (time and genotype) with Bonferroni correction. (**B**–**D**) Kruskal-Wallis test. (**A**–**D**) Representative experiment out of 2 with similar results. Scale bars: 70 μm.

To better understand the interaction of miR-21–deficient macrophages with CD8^+^T cells in the context of the TME, we performed single-cell RNA-Seq in sorted CD45^+^ cells from the tumors of *miR-21^fl/fl^* or *LysMCre;miR-21^fl/fl^* mice. We first used unsupervised clustering to generate t-distributed stochastic neighbor embedding (t-SNE) plots that separated the cells into distinct groups (Figure 11, A and B). Representation of t-SNE plots (Figure 11, overlay colored by sample) did not reveal overall outstanding differences in cell populations. The same colored cells were clustered together in the combined sample (Figure 11B). We identified and classified 7 major cell types: CD8^+^T cells, CD4^+^ T cells, NK cells, granulocytic MDSCs, monocytic MDSCs, DCs and macrophages/monocytes (see featured plots of selected marker gene overlays in Supplemental Figure 5A). Within the macrophages, 8 different clusters were detected. Based on gene markers of existing literature (57), proangiogenesis and proinflammatory macrophages were classified (Supplemental Figure 5, A and B). Within the different macrophage/monocyte clusters, the expression of canonical differentiation markers largely overlapped (Supplemental Figure 5B). All remaining macrophage cluster types failed to pass the classification threshold for any specific macrophage type and stayed as “unassigned.” Because CD8^+^ T cells were unequivocally identified, we analyzed the changes in gene expression in CD8^+^ TILs (with intact miR-21) that were either in an immune infiltrate environment mostly made up of macrophages expressing miR-21 (*miR-21^fl/fl^*) or not expressing miR-21 (*LysMCre;miR-21^fl/fl^*). Figure 11C shows a dot plot analysis of differentially expressed genes in CD8^+^T cells of the tumor immune infiltrate of *miR-21^fl/fl^* or *LysMCre;miR-21^fl/fl^* mice. Interestingly, the proportion of cells with high expression of genes associated with an activated and cytotoxic phenotype, including *Il12rb2*, *Cd69*, *Ifng*, *Ccl4*, *Ccl3*, *Cxcl10*, and several granzyme coding genes, was increased in CD8^+^ TILs of the tumor immune infiltrate of *LysMCre;miR-21^fl/fl^* mice. IPA analysis of this set of genes showed an enrichment of gene signatures that promote the communication between innate and adaptive immune cells, as well as IL-12 signaling and Th1 immune responses (Figure 11D). Prediction network analysis of these regulated genes showed IL-12 as a potential upstream regulator, while degranulation and cytotoxicity of T cells were predicted as downstream functions of these gene expression changes (Figure 11E). Importantly, blockade of IL-12 and CXCL10 in tumor-bearing *LysMCre;miR-21^fl/fl^* mice produced an increase in tumor growth as compared with *LysMCre;miR-21^fl/fl^* mice treated with an isotype control (Figure 12, A and B). Furthermore, we observed a reduction in the expression of GZMB in CD8^+^ TILs when IL-12 and CXCL10 were neutralized in *LysMCre;miR-21^fl/fl^* mice (Figure 12C), as well as an increase in neovascularization (Figure 12D). On the whole, our results provide evidence that the decreased tumor growth, improved CTL response, and decreased neovascularization observed in animals lacking miR-21 in TAMs is mediated, at least in part, by increased IL-12 and CXCL10.

### Antagonism of miR-21 in macrophages reduces tumor progression even when miR-21 is not expressed in cancer cells.

Given the desirable outcomes that miR-21 deficiency in macrophages produces, we aimed to specifically inhibit miR-21 in macrophages during tumor progression. We took advantage of a pHLIP, which is a carrier peptide that under acidic conditions can insert directionally across cell membranes and thereby translocate otherwise membrane-impermeable cargo molecules into cells via a nonendocytic route (58). Given the acidic conditions found in solid tumors, pHLIP has been shown to home to a variety of tumors when administered systemically (59). pHLIP has been coupled to antisense nucleic acid analogues consisting of peptide nucleic acids (PNAs), to create delivery vectors to successfully silence tumor miRNAs (60). The acidic environment found in the tumor has been largely attributed to the high glycolytic rate of tumor cells (61). TAMs have also activated aerobic glycolysis and thus contribute to a local acidification within the TME (62). We wondered whether pHLIP would also efficiently accumulate in TAMs in addition to tumor cells. Enhanced insertion of pHLIP into a variety of cells has been shown under acidic conditions in vitro (58). However, internalization of pHLIP by different cell types within the tumor has not been reported. Thus, we s.c. implanted eGFP-LLCs, and 2 weeks, later mice were injected with pHLIP Variant 3 (Var3) conjugated with fluorescent Alexa546 (63). Single cells of the tumor were isolated, and Alexa546 fluorescence was analyzed via flow cytometry. We found that in addition to cancer cells (eGFP LLCs), the fluorescent signal from pHLIP Var3-AF546 was also detected in TAMs (Supplemental Figure 6, A–C). Although the percentage of cancer cells that incorporated pHLIP Var3-AF546 was higher (Supplemental Figure 6A), the cell-associated fluorescence was greater in TAMs (Supplemental Figure 6, B and C). Interestingly, splenic macrophages did not show any pHLIP Var3-AF546 cell incorporation. When compared with other cells of the tumor immune infiltrate, also in an acidic microenvironment, TAMs seem to be more efficient at incorporating pHLIP Var3-AF546 because a greater number of cells showed a higher amount of inserted peptide when compared with CD4^+^ or CD8^+^ T cells and DCs (Supplemental Figure 6F). Interestingly, nonimmune cells of the TME, including ECs did not efficiently internalize pHLIP when compared with CD68^+^ macrophages within the tumor (Supplemental Figure 6F). These data indicate that pHLIP is an efficient way to target not only cancer cells but also TAMs.

**Figure 13:**
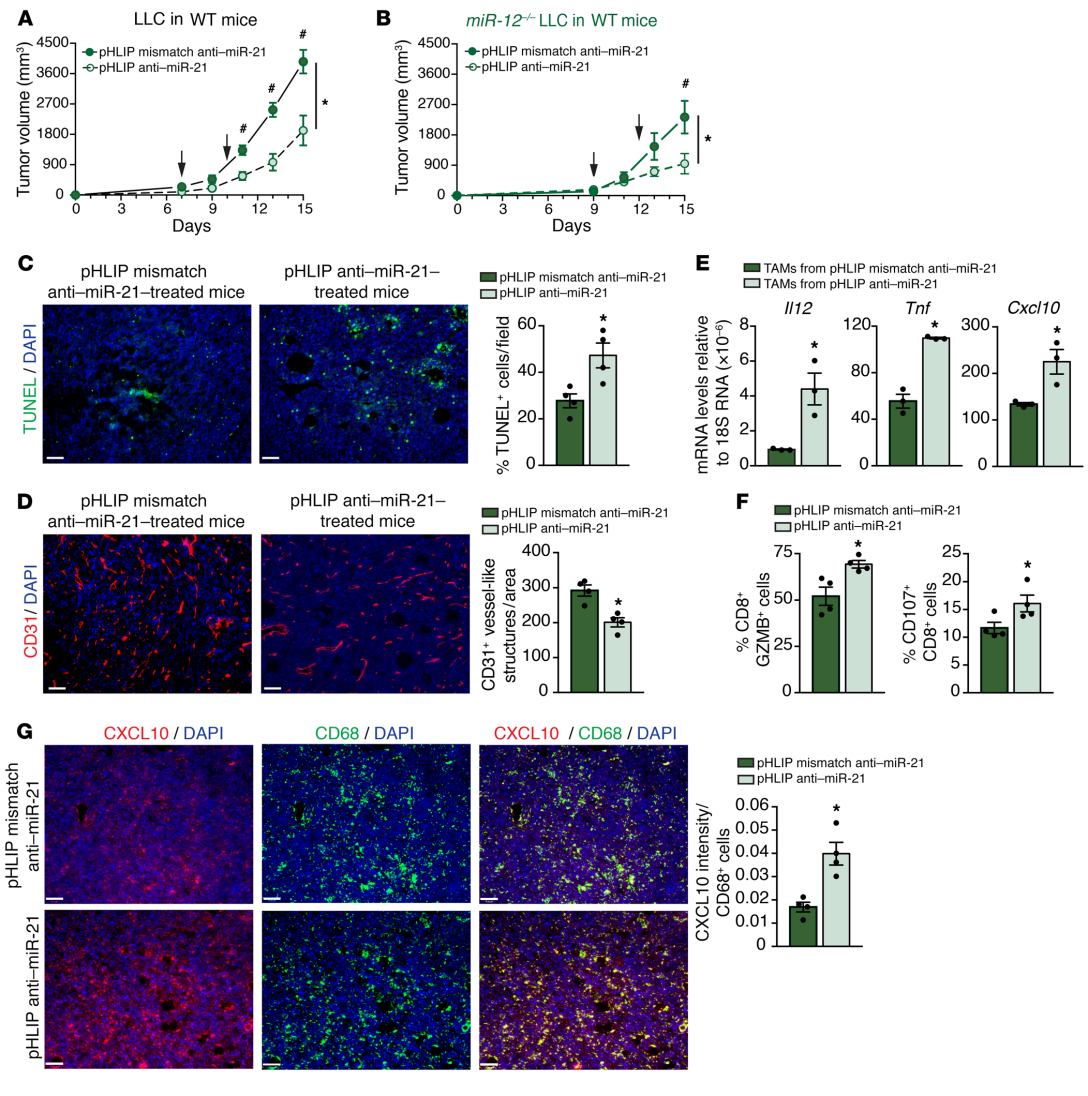
Targeting macrophage miR-21 leads to improved T-cell responses and decreased angiogenesis. (**A**) Tumor progression in *WT* mice injected s.c. with 10^6^ LLCs in the dorsal flank and treated with 1 mg/kg pHLIP anti–miR-21 or pHLIP anti–miR-21-mismatch control on indicated days (*n* = 6). (**B**) Tumor progression in *WT* mice injected s.c. with 10^6^ with *miR-21^–/–^* LLC and treated with 1 mg/kg pHLIP anti–miR-21 or pHLIP anti–miR-21-mistmatch on indicated days (*n* = 6). (**C**) Representative images of TUNEL and DAPI staining of cross sections of *miR-21^–/–^* LLC tumors of *WT* mice treated as in **B**. Right: Quantification of % DAPI^+^ TUNEL^+^ cells (*n* = 4 out of 6 randomly selected). (**D**) Representative images of CD31 staining of cross sections of *miR-21^–/–^* LLC tumors of *WT* mice treated as in **B**. Right: Quantification of CD31^+^ vessel-like structures (*n* = 4 out of 6 randomly selected). (**E**) qRT-PCR analysis of relative mRNA levels of validated or predicted targets of miR-21 (*Il12*, *Tnf*, and *Cxcl10*) in sorted TAMs (CD45^+^ CD11b^+^ MHC II^+^ F4/80^+^) of *miR-21^–/–^* LLC tumors of *WT* mice treated as in **B** (*n* = 3 out of 6 randomly selected). (**F**) Right: Flow cytometry analysis of average % of CD8^+^ TILs expressing GZMB. Left: % CD8^+^ TILs with extracellular CD107a in *miR-21^–/–^* LLC tumors of *WT* mice treated as in **B** (*n* = 4 out 6 randomly selected). (**G**) Left: Representative images of immunofluorescence costaining of CD68 and CXCL10 in frozen sections of *miR-21^–/–^* LLC tumors of *WT* mice treated as in **B**. Right: Average of quantification of CXCL10 intensity per CD68^+^ cell (*n* = 4 out 6 randomly selected). Results are mean ± SEM. *P < 0.05. (**A** and **B**) Two-way ANOVA (time and genotype) with Bonferroni correction, ^#^*P* < 0.05 individual comparisons. (**C**–**G**) Mann-Whitney *U* test. Scale bars: 70 μm.

We then assessed the therapeutic antitumor efficacy of pHLIP anti–miR-21 PNA in vivo. The PNAs were also tagged with TAMRA for visualization. Intravenous administration of pHLIP anti–miR-21 to mice with orthotopic and heterotopic lung cancer tumors resulted in a significant reduction in tumor growth when compared with mice injected with pHLIP anti–miR-mismatch control (Supplemental Figure 8, A and B, and Figure 13A). Furthermore, enhanced delivery (TAMRA fluorescence) to macrophages within the tumor was detected by flow cytometry and by immunofluorescence in CD68^+^ macrophages (Supplemental Figure 6, D and E, respectively). Despite the fact that DCs also became glycolytic upon activation (64), we only found a small proportion of DCs that showed low levels of incorporation of pHLIP Var3-AF546 (Supplemental Figure 6, A–C) or pHLIP anti–miR-21-TAMRA (Supplemental Figure 6, D and E). This was not sufficient to reduce miR-21 levels in DCs or affect miR-21 target expression (Supplemental Figure 6G).

To avoid targeting cancer cells expressing miR-21, we used CRISPR/Cas9-mediated genome editing to KO miR-21 from LLC cells (*miR-21^–/–^* LLCs) (Supplemental Figure 7). The effect of pHLIP anti–miR-21 was assessed in mice bearing *miR-21^–/–^* LLC tumors (Figure 13B). pHLIP anti–miR-21 administration still reduced tumor burden in mice bearing *miR-21^–/–^* LLC tumors, suggesting that targeting miR-21 in TAMs is sufficient to efficiently diminish tumor growth. Moreover, increased tumor cell death and reduced neovascularization were also observed (Figure 13, C and D). mRNA levels of miR-21 targets such as *Il12*, *Tnf*, or *Cxcl10* were increased in sorted TAMs from miR-21^–/–^ LLC tumors that were administered pHLIP anti–miR-21 (Figure 13E), indicating efficient miR-21 inhibition in TAMs. We also observed improved T-cell responses as indicated by increased GZMB and degranulation in CD8^+^ TILs (Figure 13F). Furthermore, increased levels of CXCL10 in CD68^+^ macrophages of these tumors was associated with reduced CD31^+^ capillary structures (Figure 13G).

Taken together, these results show that targeting miR-21 in macrophages promotes an antitumoral immune response and an overall proinflammatory angiostatic function that results in a significant reduction in tumor growth.

## Discussion

In this study, we have used a variety of different mouse models to assess how miR-21 in noncancerous cells of the TME contributes to tumor progression. Cancer initiation and progression has been historically defined by the behavior of cancer cells, including regulation by miRNAs, which have an impact on many of the autonomous functions of cancer cells to either promote or suppress tumor growth (1). Importantly, miRNAs in cells from the TME have also emerged as key players involved in the development and progression of cancer (5–7, 65).

Our results using syngeneic heterotopic and orthotopic lung cancer models demonstrate that miR-21 expression in noncancerous cells plays an important role in tumor progression, in agreement with reports using other models (24, 25). However, the existing literature fails to fully elucidate the cell compartment of the TME in which miR-21 primarily contributes to tumor progression, often relying on in vitro systems or approaches that globally target miR-21 in a non–cell/tissue-specific manner (19–25, 31).

Within the TME, miR-21 has been reported to be increased in many types of cancer (22, 27, 31, 66), and this increase was linked to tumor progression. Our data, in 3 cohorts of patients with stage I NSLC show that high expression of miR-21 in the nontumor cells of the TME is significantly associated with reduced overall survival. In agreement with these findings, a recent report shows that increased expression of miR-21 in the TME, but not in cancer cells, was associated with a poor prognosis for patients with lung adenocarcinoma (31). This effect was linked with increased expression of miR-21 in cancer-associated fibroblasts (CAFs) (31), similar to what has been reported in pancreatic and colorectal tumors (21–23). However, the underlying mechanisms of cancer-associated fibroblast–miR-21 promoting tumor growth were only investigated in vitro. We show that the expression of miR-21 in infiltrated immune cells, not stromal cells, is responsible for promoting tumor growth. This finding was supported by the decreased tumor growth in WT mice adoptively transferred with *miR-21^–/–^* BM, whereas reverse BMT (WT BM to *miR-21^–/–^* recipients) produced a converse effect. This observation was in agreement with a recent study that also used BM adoptive transplant to show that deletion of miR-21 in immune cells reduces tumor growth (25). Elevation of miR-21 levels has been observed in MDSCs directly derived from BM cells that were stimulated with GM-CSF and IL-6, as well as MDSCs from tumor-bearing mice (19), and in vitro overexpression or inhibition of miR-21 in BM cells altered MDSC expansion (19). However, within the tumor infiltrate of miR*-21^–/–^* or WT mice adoptively transferred with *miR-21^–/–^* BM, we did not find differences in the proportion of monocytic or granulocytic MDSCs when compared with controls. This finding indicates that the in vivo absence of miR-21 in MDSCs does not have a major effect on this population. Similarly, when we analyzed TILs, we did not find differences in the percentage of IFNG expressing CD4^+^ or CD8^+^ T cells, in agreement with a recent report (25). In other settings, miR-21 has been shown to regulate cell-autonomous T-cell function (67, 68). In the context of tumor progression, a report showed that absence of miR-21 reduced the proliferation of both CD4^+^ and CD8^+^ cells and their cytokine production, thus accelerating the growth of grafted tumors (24). In this case, growth of an implanted S180 mouse cancer cell line was analyzed in miR-21 floxed expressing the global Cre driver. S180 cells have been shown to grow in multiple inbred mouse strains due to β2-microglobulin deficiency, MHC class I destabilization, and impaired recognition by host CTLs (69). In this setting and without using a syngeneic mouse model and/or conditional deletion of miR-21 in T cells, it was concluded that miR-21 expression in T cells was protective against tumor growth (24).

Several studies have described correlations between macrophage number, location, and phenotypic characteristics and clinical outcomes (70). In our human TMAs, we found that high expression of miR-21 in CD68^+^ macrophages within the TME compartment had a negative impact in patient disease-specific survival and shows the relevance of miR-21 in macrophages for tumor progression. In line with miR-21 being the most abundant miRNA in macrophages (10), we found that within the LLC tumors, miR-21 is highly expressed in TAMs compared with the rest of the tumor immune infiltrate. Because conflicting results have been reported regarding the role of miR-21 in regulating the inflammatory responses of macrophages (10, 13–15, 17, 18, 43), we specifically focused on how miR-21 regulates the gene expression and functions of TAMs. miR-21 deficiency in TAMs produced a global rewiring of their transcriptional regulatory network that is skewed toward a proinflammatory phenotype and the promotion of an antitumoral immune response. Importantly, we show that selective deletion of miR-21 in macrophages in a heterotopic and orthotopic model of lung cancer mimicked the effects of global or hematopoietic loss of miR-21, with smaller tumors, increased cell death, and reduced neovascularization. Moreover, we found increased GZMB and degranulation of CD8^+^ TILs, indicating increased CTL activity and heightened killing capacity, consistent with the higher levels of cell death within the tumors of *LysMCre;miR-21^fl/fl^* mice. These findings support the report of Baer and colleagues who showed that disruption of miRNA processing in TAMs, by LysM-Cre–mediated deletion of *Dicer1*, leads to the development of smaller LLC tumors and improved CTL activity (5). This study highlights how the loss of mature miRNAs in TAMs during tumor progression leads to increased classically activated TAMs, improved CD8 response, and decreased CD8 suppression, which was associated with higher expression of CXCL10, CD86, and CXCL9 in the tumors (5). In fact, because miR-21 is highly expressed in macrophages (10), many of these changes could be attributed to the loss of expression of miR-21 after *Dicer1* deletion.

A recent report using a B16 tumor model suggested that mice receiving miR-21–deficient BM had higher numbers of “M1” TAMs with tumoricidal polarization, whereas miR-21 deficiency in T cells reduced their ability to produce proinflammatory cytokines and cytolytic granules (25). On the contrary, our data indicate that CD8^+^ TILs from *miR-21^–/–^* mice have increased GZMB expression and killing capacity. However, the increased GZMB expression is not due to miR-21 deletion in these cells because LLC tumors from *LysMCre;miR-21^fl/fl^* also showed increased levels of cell death and their CD8^+^ T cells (expressing miR-21). They also had higher levels of GZMB and extracellular CD107a, suggesting that the absence of miR-21 in TAMs can influence other cells within the TME.

Our present results indicate that the observed changes in the levels of chemokines and cytokines in miR-21–deficient TAMs are critical for CTL activation and antitumor activity. Specifically, we found that increased IL-12 in *miR-21^–/–^* TAMs is responsible for the increased GZMB levels independent of miR-21 expression in CD8^+^ T cells. This finding was supported by our single-cell RNA-Seq analysis, where we analyzed the changes in gene expression in CD8^+^ TILs from tumors in which TAMs lacked miR-21. In LLC tumors from *LysMCre;miR-21^fl/fl^* mice, the CD8^+^ TILs exhibit increased expression of genes associated with an activated and cytotoxic phenotype, including *Il12rb2*, *Cd69*, *Ifng*, *Ccl4*, *Ccl3*, *Cxcl10*, and several granzyme coding genes. These results provide evidence that the improved CTL response is the result of miR-21 deletion in macrophages.

In addition to the effects on T-cell functions, the increased levels of IL-12 in *LysMCre;miR-21^fl/fl^* mice could also play an important role in regulation of tumor angiogenesis, as IL-12 has been shown to negatively regulate tumor neovascularization (50). This effect has been associated with IFN-inducible production of CXCL10 and CXCL9 (51). Macrophages lacking miR-21 exhibited an increased production of CXCL10, which binds to the CXCR3-B receptor on endothelial cells, resulting in suppression of endothelial cell proliferation and differentiation into capillary structures (51). CXCL10 selectively attracts Th1 cells and functions as a positive feedback loop in the Th1-driven antitumor immune response (45). The tumors from *LysMCre;miR-21^fl/fl^* mice exhibited reduced neovascularization, a reduced number of proliferating TECs, and an increased percentage of TUNEL^+^ CD31^+^ TECs. Taken together, our present work indicates that the absence of miR-21 in macrophages favors the formation of a tumor- suppressive microenvironment (45), characterized by increased expression of IL-12 and CXCL10 that leads to improved CTL response, decreased neovascularization, and decreased tumor burden.

Macrophages are perhaps the most important immune cells that infiltrate the tumor and affect its pathology (71, 72). Although macrophages can show some antitumor activity (73), they mostly promote a net protumor outcome because they have been shown to be capable of dampening T-cell responses and stimulating angiogenesis, among others actions (47, 74). This phenomenon, which has been widely accepted, is promoted by tumor cells that create a widespread tolerogenic environment through the release of tumor-derived factors that promote the expansion and reprogramming of TAMs to block T-cell functions and promote several protumor events. TAMs are recognized as an important target for therapeutic intervention (75). Our data highlight the central role of miR-21 in regulation of TAM activity and function regarding CTL regulation and neovascularization. Thus, to determine whether the high expression of miR-21 in TAMs may make it a likely target for therapeutic intervention, we opted to use pHLIP anti–miR-21 PNA. pHLIP carriers coupled to antisense nucleic acid analogues are delivery vectors that have been shown to successfully target miRNAs in tumor cells (60, 76). Here, we show for the first time that TAMs incorporate pHLIP as efficiently as tumors cell and strikingly more effectively than other cells of the TME. pHLIP anti–miR-21 administration in WT mice reduced tumor burden even under conditions where tumor cells lacked miR-21 expression. Importantly, CD8^+^ TILs showed increased GZMB expression and tumors had reduced neovascularization. Sorted TAMs of pHLIP anti–miR-21 treated mice exhibited increased IL-12 and CXCL10 expression, similar to TAMs of *LysMCre;miR-21^fl/fl^* mice. Thus, targeted inhibition of miR-21 in TAMs is sufficient to increase CTL activity and decrease angiogenesis, thereby diminishing tumor growth.

Numerous studies have shown that within cancer cells, miR-21 acts as an oncomiR, and anti–miR-21 therapy (RG-012) is already in the pipeline for clinical trials. Our findings demonstrate the critical role of miR-21 within TAMs for promoting tumor development, indicating that targeting of miR-21 may have the added benefit of improving CTL activity and limiting tumor angiogenesis through its effects on TAMs. Our findings in human patients with NSCLC suggest this may have a critical impact on patient outcomes. Finally, our results indicate that use of pHLIP targeted therapies allows for effective targeting of both tumor cells and TAMs, while avoiding possible unintended effects on other tissues and organs.

## Methods

Detailed information on experimental procedures and reagents is provided in Supplemental Tables 1–3, Supplemental Figure 7, and Supplemental Methods.

### Mice.

Experiments were conducted under the ethical guidelines and protocols approved by IACUC (Institutional Animal Care and Usage Committee) in Yale University School of Medicine. WT C57BL/6 or *miR-21^–/–^* (Mir21atm1Yoli/J, Jax no. 016856) mice were purchased from The Jackson Laboratory. *miR-21^–/–^* mice were bred with C57BL/6 mice for several generations in order to ensure they were syngeneic and on C57BL/6 background. *LysMCre;miR-21^fl/fl^* on C57BL/6 background mice were generated by breeding *miR-21^fl/fl^* (*Mir21^tm1Mtm^*/Mmjax, Jax no. 36060) on C57BL/6 background with *LysMCre* (*Lyz2tm1(cre)Ifo*, Jax no. 004781); *miR-21^fl/fl^* in C57BL/6 background were used as control counterparts.

### Data and software availability.

All RNA-Seq and single-cell RNA-Seq data were deposited in the NCBI’s Gene Expression Omnibus database (GSE117697 and GSE118931).

### Statistics.

Data are presented as mean ± SEM. Statistical differences were measured using 1-way ANOVA or 2-way ANOVA with Bonferroni correction for multiple comparisons. Normality was checked using the Kolmogorov–Smirnov test. A nonparametric test, Mann-Whitney *U*, test or a Kruskal-Wallis test for multiple comparisons, was used when data did not pass the normality test. For RNA-Seq analysis, we used Benjamini-Hochberg False Discovery Rate. Survival curves were generated by Kaplan–Meier analysis and tested for significance using the Mantel-Cox log-rank test. A value of *P* ≤ 0.05 was considered statistically significant. Data analysis was performed using GraphPad Prism Software Version 7.

### Study approval.

Animal experiments were conducted under the ethical guidelines and protocols approved by IACUC at Yale University School of Medicine (Animal protocol no. 2019-116576). All human tissues were collected with the approval from the Yale Human Investigation Committee (protocol no. 9505008219). The Yale Human Investigation Committee approved the patient consent forms or in some cases, a waiver of consent.

## Author contributions

MS, BC, and JS assisted with experimental design, performed experiments, analyzed data, and interpreted results. YL, RGM, and SM analyzed data. WD performed experiments. YKR provided pHLIP (Var3). AK, SO, and RB synthesized miR-21 PNA and conjugated to pHLIP. NLP analyzed data and edited the manuscript. PF provided *LysMCre;miR-21^fl/fl^* mice. PMG, DLR, and CF-H assisted with experimental design and data interpretation. YS designed the study, analyzed data, interpreted results, and wrote the manuscript (comments were added by all authors).

## Supplementary Material

Supplemental data

## Version 1 11/11/2019

Electronic publication

## Version 2 12/02/2019

Print issue publication
