# Supplementary material for: Suppressing miR-21 activity in tumor-associated macrophages promotes an antitumor immune response
Source: J Clin Invest. 2019 Nov 11;129(12):5518–36. doi: 10.1172/JCI127125 (PMC6877327; doi:10.1172/JCI127125)
Supplement: Supplemental data [file jci-129-127125-s304.pdf]

Supplemental Data

Suppressing miR-21 Activity in Tumor Associated Macrophages Promotes an Antitumor Immune Response

Mahnaz Sharaei, Balkrishna Chaube, Yuting Liu, Jonathan Sun, Alanna Kaplan, Nathan L Price, Wen Ding, Stanley Oyaghire, Rolando García-Milian, Sameet Mehta, Yana K Reshetnyak, Raman Bahal, Paolo Fiorina, Peter M Glazer, David L Rimm, Carlos Fernández-Hernando and Yajaira Suárez

Supplemental Methods

Murine Syngeneic Tumor models

Heterotopic model of Lewis lung cancer (LLC) and orthotopic B16 mouse melanoma were used. For orthotopic model of lung cancer LL/2 Red-Fluc cells (Perkin Elmer, Hopkinton, MA) were used. LLC (LL/2 (LLC1-ATCC CRL-1642) and B16 (B16-F0-ATCC CRL-6322) cells were grown exponentially. 10^6 cells were injected subcutaneously into the dorsal flank of 8-10-week-old mice as described (1). When tumors became palpable, they were monitored for growth by measuring the length and width of the tumor using a caliper, and tumor volume was determined by the following formula: volume=0.52×(width)^2×(length). Mice with no palpable mass at day 8 were removed from the study.

Unless otherwise indicated, after 14 days, the animals were euthanized and tumor tissues were collected and weighted. Tumors were then cut into pieces and immediately embedded and frozen in OCT compound for subsequently immunofluorescence or digested to obtain single cell suspension for flow cytometry or FACS sorting analysis. In some instances, animals were implanted s.c. as above indicated with LLC constitutively expressing eGFP (eGFP-LLC) or LLC which expression of miR-21 was knocked out using CRISPR/Cas9 approach (*miR-21*$^{-/-}$ LLCs), see below for details generation of these stable LLC lines.

For orthotopic model of lung cancer 5x10^5 LL/2 Red-Fluc cells were injected into the upper margin of the sixth intercostal rib on the right anterior axillary line. For visualization and estimation of tumor growth, mice were anesthetized with isoflurane inhalation and injected i.p. with 150 mg/kg XenoLight D-Luciferin - K+ (Perkin Elmer, Hopkinton, MA) dissolved in DPBS 10 min before bio-luminescence imaging that was performed with a CCD camera (IVIS spectrum, PerkinElmer). Bioluminescent signals were quantified using Living Image 3.4

(Caliper Life Sciences, Alameda, USA) on every fourth day. Luminescence from the region of interest (ROI) was defined manually, and the data were expressed as photon-flux (photons/s/cm^2/steradian). Mice that did not show tumor growth at day 8 were removed from the study.

Bone Marrow Adoptive Transplant

6-8-week-old *WT* or *miR-21*$^{-/-}$ mice were lethally irradiated twice with a dose of 550 rads (5.5 Gy) using a cesium source 4 hours before transplantation, as described (2). Bone marrow was collected from the femur and tibia of WT or *miR-21*$^{-/-}$ mice with sterile PBS and RBCs were lysed using a RBC lysis buffer (BD, Franklin Lakes, NJ). Cells (2×10^6) were re-suspended in sterile saline and retro-orbitally injected into irradiated recipient mice. Mice were administered with antibiotics in their water for two weeks followed by two weeks of recovery. Four weeks after bone marrow transplantation (BMT), peripheral blood was collected by retro-orbital venous plexus puncture for qRT-PCR analysis of miR-21 for bone marrow reconstitution. Heterotopic model of Lewis lung cancer was used for these experiments as described above.

Immunofluorescence and TUNEL staining on frozen sections

Tumors were excised and necrotic areas were trimmed and excluded from the preparation. Dissected tumors were cut into 5mm pieces that represent different areas of the tumor and fixed in 4% PFA on ice for 4hrs. Tumors were then incubated in 30% sucrose solution overnight and then embedded in OCT. Sections (4-6 um) were mounted on poly lysine coated slides (Thermo Fisher, Waltham, MA). Slides were permeabilized 10 minutes with 0.1% Tween in PBS and blocked in 20% FBS in PBS. For immunostaining, anti-CD31-PE fluorescently labeled antibody (clone:MEC13.3,BD, Franklin Lakes, NJ) was used to detect vessel structures, anti-CXCL10 antibody (clone: AF-466-NA R&D, Minneapolis, MN) was used to detect CXCL10 in tumors and together with anti-CD68 antibody (Clone:FA-11, Biorad, Hercules, CA) was used as a macrophage marker. CXCL10 and CD68 were detected using donkey AF-488 conjugated donkey anti rat antibody (Invitrogen, Carlsbad, CA cat # A-11055) and AF-647 conjugated goat anti-rat antibody (Invitrogen, Carlsbad, CA cat # A-21247) accordingly. Apoptotic cells in tumor sections were detected by TUNEL after proteinase K treatment, using the In-Situ Cell Death Detection kit, TMR red (Roche, Basel, Switzerland) according to the manufacturer's instructions. Nuclei were counterstained using ProLong gold containing DAPI (Invitrogen, Carlsbad, CA). Fluorescent-labeled samples were visualized with a Zeiss Axiovert

2000M fluorescence microscope. Images were acquired with a CCD camera (Axio; Zeiss MicroImaging). For quantification, three sections per sample were analyzed and from each sample two to four images were captured from random areas of each tumor section. Microvessel density was quantified by measuring the CD31$^+$ vessel-like structures per sample area as described previously (3, 4). At least two areas per tumor were analyzed, three sections per sample were quantified and from each sample two images were captured from random areas of each tissue section. ImageJ was used to determine the number of positive structures per sample area. CXCL10 intensity was expressed per CD68$^+$cell per section area. The values per area of individual images were then averaged to obtain the mean value per area-fraction for each tumor. NIH ImageJ software (National Institutes of Health, Bethesda, MD, USA) was used for all the quantifications.

Analysis of tumor immune infiltrate

Isolation of tumor infiltrating immune cells was done as described previously (5) . Briefly, tumors were excised and necrotic areas were trimmed and excluded from the preparation. Tumors were cut into 3mm pieces from different areas of the tumor and digested in 0.5mg/ml collagenase III (Worthington, Lakewood, NJ) at 37°C for 20 minutes. Digested tissue was passed through 40 µm strainer and treated with DNase I (Sigma, St. Louis, MO) for 10 minutes in room temperature. After several washes, cells were used for flow cytometry analysis or single cell sorting as described below. For cytokine secretion studies cells were incubated for 4hrs with solution of Mononesin and Brefeldin A in 37°C following manufacturer's instruction (Biolegend, San Diego, CA) prior antibody incubation and flow cytometry analysis.

Flow cytometry analysis and single cell sorting of tumor immune infiltrate.

The following antibodies were used for flow cytometric analyses or single cell sorting: F4/80, Clone:BM8; CD45, Clone:30-F11; Ly6C, Clone:HK1.4; CD11b, Clone:M1/70; Ly6G, Clone:1A8; TNFa, Clone:MP6-XT22; Ki67, Clone:6A8; CD86, Clone:53-5.8; CD86, Clone:GL-1; CD40 3/23; CD3, Clone:145-2C11; CD8a, Clone:53-6.7 were purchased from Biolegend (San Diego, CA), IL12, Clone:C17.8; MHCII, Clone:M5/114.15.2; CD107a, Clone:eBio1D4B; pIL-1b, Clone:NJTEN3; IFNg, Clone: XMG1.2 were obtained from eBioscience (Cupertino, CA), CD31, Clone:MEC13.3; CD11c, Clone:HL3; GZMB, Clone:GB11 were from BD (Franklin Lakes, NJ), CCR2, Clone:FABS538A was from R&D (Minneapolis, MN), CD68, Clone:FA-11 was purchased from BioRad (Hercules, CA). We used a 1:200

dilution for all antibodies. All antibodies were titrated to determine optimal signal-to-noise separation and minimize background fluorescence. Gating strategy was set up using Fluorescence Minus One (FMO) control. Tumor associated macrophages (TAMs) were identified and sorted as $CD45^{+}CD11b^{+}MHCII^{+}$ $F4/80^{+hi}$, $CD4^{+}$ T-cells identified as: $CD45^{+}CD4^{+}$, $CD8^{+}$ T cells identified as: $CD45^{+}CD8^{+}$, MDSCs identified as: $CD45^{+}CD11b^{+}Gr1^{+}Ly6C^{+}LY6G^{+}$, dendritic cells (DCs) were identified and sorted as $CD45^{+}CD11b^{+}$ $Cd11c^{+}$ $MHCII^{+}$ $F4/80^{-}$, tumor endothelial cells (TECs) identified as: $CD45^{-}CD31^{+}$. Apoptotic cells were analyzed with fluorescein isothiocyanate (FITC)-conjugated annexin V staining (1:20, Biolegend) together with propidium iodide (PI) dead cell counterstain according to the manufacturer's recommendations) and as described previously (2). All flow cytometric analyses were performed using a BD LSRII (BD Biosciences). All flow cytometry data were analyzed using FlowJo software v8.8.6 (Tree Star, Inc.). Data of the analyzed immune cell types are shown as percentage $CD45^{+}$ cells (mean values ± SEM). In some instances, digested cells from the tumor were sorted on a BD FACS Aria (BD Biosciences).

Patient cohort and tissue microarray

Three retrospective NSCLC cohorts termed YTMA79, YTMA140 and YTMA250 were used in this study. YTMA140 includes samples from 344 NSCLC patients, collected by Sotiria General Hospital and Patras University General Hospital (Greece) between 1991-2001. YTMA79 comprises a sample collection from 178 NSCLC between 1988-2003 at Yale Pathology. YTMA250 comprises a surgical resection sample collection from 263 NSCLC patients between 2004-2011 at Yale Pathology. All patient samples were formalin-fixed, paraffin-embedded (FFPE) and were prepared as a tissue microarray (TMA) as previously described (6). With pathology review, representative tumor areas were chosen from the samples and cored with a needle at 0.6mm to make a recipient FFPE TMA block. Due to loss of tissue, unavailable survival data and/or poor quality of the staining, fewer cases were finally included in the analysis (87 out of 178 for YTMA79; 95 out of 344 for YTMA140; 128 out of 263 available for YTMA250). Clinicopathologic characteristics of this cohort can be found in Supplemental Tables S1, S2 and S3.

miRNA *in situ* hybridization and quantitative immunofluorescence analysis

miR-21, CD68, and cytokeratin were detected on human lung cancer tissues of Yale tissue microarrays (TMA) using AQUA technology as described (7-10). In brief, formalin-fixed

paraffin-embedded TMAs were deparaffinized in three exchanges of xylene, followed by rehydration in an ethanol gradient. Antigen retrieval was performed by incubating slides in a solution of EDTA and sodium hydroxide at 97°C for 30 minutes. Endogenous peroxidases are blocked in 1% H_2O_2 for 30 min. Overnight primary antibodies were added after blocking in a BSA solution, followed by secondary staining. HuCD68 (clone:PG-M1, Agilent Santa Clara, CA) was used to detect macrophages followed by AF-647 conjugated goat anti-mouse antibody (Invitrogen, Carlsbad, CA cat # A21235). To detect tumor epithelial cells, anti-Cytokeratin Polyclonal antibody (Agilent, Santa Clara, CA) was used followed by AF-488 conjugated goat anti rabbit antibody (Invitrogen, Carlsbad, CA cat # A-11011). Then slides rinsed twice in 0.13M 1-methylimidazole (Sigma-Aldrich, St Louis, MO, USA) and fixed with 1-ethyl-3-(3-dimethylamino propyl) carbodiimide (EDC, Thermo Scientific) as described previously (10). Slides were then prehybridized at the hybridization temperature (53°C) for 30 min in hybridization buffer with 500 mg/ml yeast tRNA (Invitrogen, Carlsbad, CA, USA), 50% formamide(American Bioanalytical, Natick, MA, USA), 5 SSC (American Bioanalytical), 50 mg/ml Heparin (Sigma-Aldrich), 0.1% Tween-20 (Sigma-Aldrich) at pH6. Slides are hybridized for 1 h with 100nM double Digoxigenin (DIG) LNA-modified probes (Cat number: 38102-15, EXIQON, Qiagen, Hilden, Germany) at 53°C for 1hr. After several rounds of stringency washes with 2XSSC, slides were blocked with 2% BSA (Sigma-Aldrich) for 30 min and incubated with anti-DIG-POD, Fab fragments from sheep (Roche Diagnostics) diluted 1:100 to detect miR-21. To detect Dig labeled antibody, TSA plus Cy5 kit (Perkin Elmer, Waltham, MA) was utilized according to the manufacturer's instructions.

Quantitative immunofluorescence was quantified using the Vectra Polaris Automated Quantitative Pathology Imaging System (Perkin Elmer®). Multispectral images were obtained in all channels. With trainable feature-recognition 5nform software, cell types and tissue types were identified. The cell count was calculated with the trained cell types of interest. Spots with less than 2% of tumor were excluded from the final analysis.

RNA isolation and qRT-PCR analysis.

Total RNA was isolated using TRIzol reagent (Invitrogen) and Zymo Research RNA isolation kit according to the manufacturer's instructions and was used for detection of mRNA and miRNA levels. 0.5-1ug of total RNA was reverse transcribed using the iScript RT Supermix (BioRad, Hercules, CA), following the manufacturer's protocol. qRT-PCR was performed in

triplicate using SoFast EvaGreen Supermix (BioRad) on a Real-Time Detection System (Bio-Rad, Hercules, CA). The mRNA levels were normalized to 18S RNA as a housekeeping gene. Primers used for qRT-PCR were: *tnf*: Forward: CCCTCACACTCAGATCATCTTCT, reverse: GCTACGACGTGGGCTACAG, *Il12a*: Forward: TGGTGAAGACGGCCAGAGAA, reverse: GGCACAGGGTCATCATCAAA. *Pten*: forward: CTGGGCTTAAGGTCTGATTCTC, reverse: CAGTGAATGCCATCACCATTTC. *Cxcl10*: forward: CTGAGTGGGACTCAAGGGAT reverse: GTGGCAATGATCTCAACACG. *18S*, 5'-TTCCGATAACGAACGAGACTCT-3' and 5'-TGGCTGAACGCCACTTGTC-3'.

Mature miR-21 levels (miR-21) were detected using TaqMan miRNA Assay kit (Life Technologies, Carlsbad, CA) following the manufacturer's protocol as described (2, 4). qRT-PCR was performed using TaqMan Universal Master Mix (Life Technologies). RNA U6 was used for normalization. Primary transcript-miRNA (Pri-miR-21) levels were detected using the TaqMan RT kit (Life Technologies) according to manufacturer's protocol (4). Processed, precursor miRNA (Pre-miR-21) levels were detected using miScript Pre-cursor Assay kit (Qiagen) according to the kit's protocol.

RNA-sequencing

Total RNA from sorted TAMs was isolated using Qiagen's RNeasy Micro kit. The purity and integrity of total RNA samples was verified using the Agilent Bioanalyzer (Agilent Technologies, Santa Clara, CA at Yale Center for Genomic Analysis (YCGA). 500ng of total RNA was purified with oligo-dT beads. The cDNA library is then end-repaired, and A-tailed using Kapa mRNA Hyper prep kit (Cat# KK8581, Wilmington, MA). Indexed libraries that meet appropriate cut-offs for both are quantified by qRT-PCR using a commercially available kit from Kapa DI Adapter Kit (Cat# KK8722, Wilmington, MA) and insert size distribution determined with the LabChip GX or Agilent Bioanalyzer. Samples are sequenced using 75 bp single or paired-end sequencing on an Illumina HiSeq 2500 according to Illumina protocols. Primary analysis - sample de-multiplexing and alignment to the human genome - was performed using Illumina's CASAVA 1.8.2 software suite.

Analysis of RNA-sequencing data and pathway analysis

The RNA-sequencing and statistical analysis was performed using Partek Flow software version 7.0, build 7.18.0130. Copyright ©; 2018 Partek Inc., St. Louis, MO, USA. Paired-end reads were trimmed using a base quality score threshold of >20 and aligned to the Genome

Reference Consortium Mouse Build 38 (mm10) with the STAR aligner. Total counts per gene were quantified and normalized to identify differentially expressed genes (DEGs). FASTQ and processed data are available at Gene Expression Omnibus (**GSE117697**). Qlucore Omics Explorer 3.4 (Qlucore AB, Lund, Sweden) was used for hierarchical clustering and heatmap generation, and PCA of log2 transformed of global expression values. DEGs used in pathway analysis were determined between KO and WT by using a filtering criteria fold change (FC) > 1.5 and $P \leq 0.005$, Benjamini-Hochberg False Discovery Rate (B-H FDR) $P \leq 0.1$ (11). Ingenuity Pathway Analysis (QIAGEN Redwood City, USA, www.qiagen.com/ingenuity), Content version:45865156 was used to identify specific pathways and diseases and functions overrepresented in the DEG.

miRNA target prediction analysis

Mouse miRNA target predictions were obtained from miRwalk (12). Only targets predicted by at least two out of four algorithms were chosen for further analysis. Venny 2.0.2 [Oliveros, J.C. (2007-2015) http://bioinfogp.cnb.csic.es/tools/venny/index.html] was also used to find targets which mRNA levels were significantly increase (FC>1.5, $P \leq 0.005$ B-H FDR) in *miR-21*$^{-/-}$ TAMs vs. *WT* TAMs.

BMDM isolation and culture

Bone marrow-derived macrophages (BMDMs) from adult male WT or *miR-21-/-* mice were harvested and cultured in Iscove's Modified Dulbecco's medium (IMDM) supplemented with 20% fetal bovine serum and 20% L-929 cell–conditioned medium as we have described (2). After 7 days in culture, non-adherent cells were eliminated, and adherent cells were harvested and plated for described experiments. BMDMs from WT and *miR-21*$^{-/-}$ mice were used to determine the effect on inflammatory cytokines. Briefly, BMDMs were stimulated for 6h with LPS (10ng/ml) (Sigma). At the end of the treatment, cells were extensively washed with PBS and RNA was isolated for analysis of mRNA levels as described above.

CTL assay

$CD8^+$ T cells ($CD45^+$, $CD8^+$) were FACs sorted from LLC tumors of *WT* or *miR-21*$^{-/-}$and incubated with LLCs in a 1:1 or 2:1 effector: target ratio overnight. The next day LLCs were counted using a hemocytometer. Viability was determined by Trypan Blue exclusion. Apoptotic LLCs were analyzed by flow cytometry as described above.

T-cell activation assay

CD8$^+$ T cells were sorted from the spleen of healthy 6-8 weeks old *WT* mice using anti-CD8a antibody (Biolegend, San Diego, CA) bound Dynabeads (Invitrogen, Carlsbad, CA) according to the manufacturer's protocol. Cells were incubated in DMEM containing 10% FBS supplemented with pen/strep and L-glutamine (Gibco, Gaithersburg, MD) for 3-5 days depending on the assay on anti-CD3 Antibody (Biolegend, San Diego, CA) bound plates (5µg/ml). At the end of the incubation period, cells were harvested and GZMB levels were determined by flow cytometry. In some experiments, *WT* splenic CD8$^+$ T cells were incubated in conditioned media obtained from TAMs isolated from LLC tumors of *WT* of *miR-21*$^{-/-}$ mice. To determine the involvement of IL12 TAM, conditioned media was treated with 20µg/ml of neutralizing IL12 antibody (clone:C17.8, Biolegend, San Diego, CA) or rat IgG isotype antibody (Biolegend, San Diego, CA).

Conditioned media from TAMs in culture

TAMs were FACS sorted from the LLC tumors of *WT* of *miR-21*$^{-/-}$ mice as described above. After sorting, TAMs were counted and live cells were cultured overnight in DMEM containing 10% FBS at a concentration of 10^5 cells/0.2ml. The next day, supernatant was collected, spun at 12000rpm at 4° C for 10 minutes and added to day 3 activated WT splenic CD8$^+$ T cells.

Single Cell RNA-sequencing

Total CD45$^+$ immune cells were FACS sorted from the LLC tumors of *LysMCre;miR-21*$^{f/f}$ or *miR-21*$^{f/f}$ mice (n=3), counted and assessed for viability with Trypan Blue using a Countess II automated counter (Invitrogen, Carlsbad, CA), and adjusted at a concentration of 800-1000 cells/μL. Final cell viability estimates ranged between 80-93%. Single cell suspensions were loaded onto 10X Genomics Single Cell A Chips (10x Genomics, PN-120236) along with the reverse transcription (RT) master mix as per the manufacturer's protocol for the Chromium Single Cell 3' Reagent kits V2. (10X Genomics; PN-120237), to generate single cell gel beads in emulsion (GEMs) and sequencing libraries. Libraries were sequenced on an Illumina HiSeq2500 using rapid mode as follows: 26 bp (Read 1); 8bp (i7 Sample Index); 0bp (i5 Index); 98bp (Reads 2). Processing of the sequencing data into transcript count tables was performed using the Cell Ranger Single Cell Software Suite 2.1.0 by 10X Genomics (http://10xgenomics.com/). Data are available at Gene Expression Omnibus (**GSE118931**)

Single Cell RNA-sequencing analysis

The sequencing data was processed using cellranger with default parameters and appropriate reference genome and annotation, in this case mm10. The output from the cellranger was processed using Seurat as described (http://satijalab. org/seurat/). Default cut-offs were used for detection of cells and genes. Specifically, a cell was considered to be a "real" cell if and only if it had at least 200 genes expressed. Similarly, a gene was considered to be "expressed" if and only if it was expressed in at least 3 cells. The cells with more than 10% of mitochondrial transcripts were eliminated, similarly cells that showed more than 3000 detected genes were eliminated as these are potentially doublets. For the rest of the cells and transcripts the numbers were normalized to about 10000 transcripts per cell, and log normalized. The variable genes were detected using Seurat, and were used for clustering using PCA. The clusters were rendered using tSNE, and cluster markers were obtained using Seurat default settings.

Neutralizing IL12 and CXCL10

8 week old *LysMCre;miR-21*$^{f/f}$ or *miR-21*$^{f/f}$ mice that were injected subcutaneously in the dorsal flank with LLCs. At days 8, 11 and 13 mice were injected intraperitoneally with 200µg of anti-IL12 (Clone: 17.8, Biolegend, San Diego, CA) + 100µg of anti-CXCL10 (Clone: 134013m Invitrogen, Carlsbad, CA) neutralizing antibodies. Control mice were injected with 300µg isotype control antibody (Biolegend, San Diego, CA). Tumor progression was measured throughout the study as explained previously. At day 15 tumors were harvested for FACS and immunofluorescence analysis as described above.

pHLIP Var3 cell targeting analysis

eGFPs-LLCs were injected in the flank of *WT* mice. At day 12 post tumor cell injection mice were retro-orbitally injected with fluorescently labeled (alexa Fluor 5546) pHLIP Var3 peptide (AF546~pHLIP Var 3) (13). Four hours post injection, tumors were dissected and digested as described above. The uptake of AF546~pHLIP Var3 was measured by flow cytometry in eGFP-LLC (eGFP$^+$ cells) , as well as in different cells of the immune infiltrate including TAMs, CD8$^+$ T cells , CD4$^+$ T cells and DCs using the markers and antibody panel described above. The results are expressed in terms of mean intensity of fluorescence (M.I.F.) after subtracting the fluorescence of cells that were obtained from PBS injected mice.

PNA Synthesis and pHLIP-PNA conjugation

PNA synthesis was performed as previously described (14) using standard Boc chemistry procedures on a solid-support. To facilitate linkage of pHLIP to PNAs, cysteine was conjugated to the C-terminus of PNAs using a Boc-miniPEG-3 linker, denoted in the sequences by –ooo- (11-Amino-3,6,9-Trioxaundecanoic Acid, DCHA, Peptide International). Single isomer 5-Carboxytetramethylrhodamine (TAMRA, Thermo Fisher) was also conjugated to the N-terminus of PNAs using a Boc-miniPEG-3 linker. PNA oligomers were cleaved from the resin using a cocktail solution consisting of m-cresol: dimethylsulfide (DMS):TFMSA:TFA (1:1:2:6), precipitated with ether, and purified by HPLC and characterized using MALDI-TOF. The following PNA sequences were used: Anti-miR-21: TAMRA-OOO-TCAACATCAGTCTGATAAGCTA-OOO-Cys**.** Mismatch: TAMRA-OOO-ATCCATAACCATGCGATGTAAT-OOO-Cys. pHLIP-PNA conjugations were performed as previously described (15). Briefly, pHLIP-Cys(NPys) (AAEQNPIYWARYADWLFTTPLLLLDLALLVDADEGT(CNPys)G, (New England Peptide) and PNAs were reacted overnight at a 1:1.2 ratio in the dark in a mixture of DMSO:DMF:0.1mM KH2PO4 pH 4.5 (3:1:1). Further, pHLIP-PNA conjugates were purified by RT-HPLC analysis and characterized using MALDI-TOF.

pHLIP-anti-miR-21 treatment

8 week old *WT* mice that were injected subcutaneously in the dorsal flank with either LLCs or *miR-21*$^{-/-}$ LLCs. Mice that were implanted with miR-21-expressing LLCs were injected intravenously with 1mg/kg of pHLIP-anti-miR-21-TAMRA or pHLIP-mismatch control (15) on day 7, when the tumor mass became palpable and subsequently at day 10. Mice that were implanted with non-expressing *miR-21*$^{-/-}$ LLCs, were injected with 1mg/kg of pHLIP-anti-miR-21 or pHLIP-mismatch control (15) on day 9 post tumor injection and at day 12. Tumor progression was measured throughout the study as explained previously.

***miR-21*$^{-/-}$ LLCs generation**

To introduce double strand breaks into the miR-21 gene we used CRISPR/Cas9 technology as described (16). Briefly, A gRNA was designed using http://crispr.mit.edu/ against the PAM sequence upstream of the mature miR-21 loop with the following sequence: CACCGATGTTGACTGTTGAATCTCA (Supplemental Figure S7A). Targeting the hairpin loop is critical for Dicer dependent pre-miRNA processing (17) and has been show to efficiently disrupt miR-21 expression in cancer cell lines (18). Following insertion of the gRNA to the

pSpCas9(BB)-2A-GFP plasmid (Adgene, Cambridge, MA), LLCs were transfected overnight using lipofectamine (Life Technologies). Single GFP^{+} cells were FACs sorted and cultured as single cell per well in 96 well plate. Deletion of 3 nucleotides in the loop region of miR-21 gene was detected by PCR amplification of genomic DNA using Forward: TGTTGTTGGGCTGGAGATCT and reverse: CCAAAATGTCAGACAGCCCA and subsequent sequencing. miR-21 levels in *miR-21*$^{-/-}$ LLC mutated clone was determined by qRT-PCR as described above. A significant decrease in expression of mature miR-21 was observed when compared to control LLCs (Supplemental Figure S7B). The levels of the primary transcripts of miR-21 (pri-miR-21) were not affected (Supplemental Figure S7B). Interestingly, the level precursor pre-miR-21 were increased (Supplemental Figure. S7B) which is indicative of an inefficient processing by Dicer (17). As expected, *miR-21*$^{-/-}$ LLCs showed increased expression of *Pten* (a known miR-21 target) (Supplemental Figure S7C). Transfection with miR-21 mimic reduced its expression, whereas, transfection with miR-21 inhibitor did not alter the expression of PTEN in *miR-21*$^{-/-}$ LLCs when compared to control counterparts (Supplemental Figure. S3C) indicating that these cells have an efficient inactivation of miR-21. *miR-21*$^{-/-}$ LLCs also had decreased proliferation (18) when compared to control LLCs (Supplemental Figure S7D). Transfection with miR-21 mimic (see below) rescued this effect (Supplemental Figure S7D). Whereas, transfection with miR-21 inhibitor did not alter the decreased proliferation of *miR-21*$^{-/-}$ LLCs (Supplemental Figure S7D).

miR-21 mimic and inhibitor transfection

LLCs were transfected with 40 nM miR-21 mimic or 60 nM miR-21 inhibitor (Dharmacon, Lafayette, CO) using RNAiMAX (Invitrogen, Carlsbad, CA) for 8 h, as described previously (2, 4). All experimental control samples were treated with an equal concentration of a non-targeting control mimic or inhibitor sequence. 48-72 hours post-transfection, cells were collected and counted to determine the effect on proliferation, or harvested for mRNA analysis.

Supplemental References for Supplemental Data

1. Suarez Y, Fernandez-Hernando C, Yu J, Gerber SA, Harrison KD, Pober JS, Iruela-Arispe ML, Merkenschlager M, and Sessa WC. Dicer-dependent endothelial microRNAs are necessary for postnatal angiogenesis. *Proc Natl Acad Sci U S A*. 2008;105(37):14082-7.
2. Canfran-Duque A, Rotllan N, Zhang X, Fernandez-Fuertes M, Ramirez-Hidalgo C, Araldi E, Daimiel L, Busto R, Fernandez-Hernando C, and Suarez Y. Macrophage deficiency of miR-21 promotes apoptosis, plaque necrosis, and vascular inflammation during atherogenesis. *EMBO Mol Med*. 2017;9(9):1244-62.
3. Cantelmo AR, Conradi LC, Brajic A, Goveia J, Kalucka J, Pircher A, Chaturvedi P, Hol J, Thienpont B, Teuwen LA, et al. Inhibition of the Glycolytic Activator PFKFB3 in Endothelium Induces Tumor Vessel Normalization, Impairs Metastasis, and Improves Chemotherapy. *Cancer Cell*. 2016;30(6):968-85.
4. Chamorro-Jorganes A, Lee MY, Araldi E, Landskroner-Eiger S, Fernandez-Fuertes M, Sahraei M, Quiles Del Rey M, van Solingen C, Yu J, Fernandez-Hernando C, et al. VEGF-Induced Expression of miR-17-92 Cluster in Endothelial Cells Is Mediated by ERK/ELK1 Activation and Regulates Angiogenesis. *Circ Res*. 2016;118(1):38-47.
5. Tinder TL, Subramani DB, Basu GD, Bradley JM, Schettini J, Million A, Skaar T, and Mukherjee P. MUC1 enhances tumor progression and contributes toward immunosuppression in a mouse model of spontaneous pancreatic adenocarcinoma. *J Immunol*. 2008;181(5):3116-25.
6. McCabe A, Dolled-Filhart M, Camp RL, and Rimm DL. Automated quantitative analysis (AQUA) of in situ protein expression, antibody concentration, and prognosis. *J Natl Cancer Inst*. 2005;97(24):1808-15.
7. Camp RL, Chung GG, and Rimm DL. Automated subcellular localization and quantification of protein expression in tissue microarrays. *Nat Med*. 2002;8(11):1323-7.
8. Anagnostou VK, Dimou AT, Botsis T, Killiam EJ, Gustavson MD, Homer RJ, Boffa D, Zolota V, Dougenis D, Tanoue L, et al. Molecular classification of nonsmall cell lung cancer using a 4-protein quantitative assay. *Cancer*. 2012;118(6):1607-18.
9. Hanna JA, Wimberly H, Kumar S, Slack F, Agarwal S, and Rimm DL. Quantitative analysis of microRNAs in tissue microarrays by in situ hybridization. *Biotechniques*. 2012;52(4):235-45.
10. Hanna JA, Hahn L, Agarwal S, and Rimm DL. In situ measurement of miR-205 in malignant melanoma tissue supports its role as a tumor suppressor microRNA. *Lab Invest*. 2012;92(10):1390-7.
11. Benjamini Y, and Hochberg Y. Controlling the False Discovery Rate - a Practical and Powerful Approach to Multiple Testing. *J Roy Stat Soc B Met*. 1995;57(1):289-300.
12. Dweep H, Sticht C, Pandey P, and Gretz N. miRWalk--database: prediction of possible miRNA binding sites by "walking" the genes of three genomes. *J Biomed Inform*. 2011;44(5):839-47.
13. Tapmeier TT, Moshnikova A, Beech J, Allen D, Kinchesh P, Smart S, Harris A, McIntyre A, Engelman DM, Andreev OA, et al. The pH low insertion peptide pHLIP Variant 3 as a novel marker of acidic malignant lesions. *Proc Natl Acad Sci U S A*. 2015;112(31):9710-5.

14. Christensen L, Fitzpatrick R, Gildea B, Petersen KH, Hansen HF, Koch T, Egholm M, Buchardt O, Nielsen PE, Coull J, et al. Solid-phase synthesis of peptide nucleic acids. *J Pept Sci*. 1995;1(3):175-83.
15. Cheng CJ, Bahal R, Babar IA, Pincus Z, Barrera F, Liu C, Svoronos A, Braddock DT, Glazer PM, Engelman DM, et al. MicroRNA silencing for cancer therapy targeted to the tumour microenvironment. *Nature*. 2015;518(7537):107-10.
16. Ran FA, Hsu PD, Wright J, Agarwala V, Scott DA, and Zhang F. Genome engineering using the CRISPR-Cas9 system. *Nat Protoc*. 2013;8(11):2281-308.
17. Gu S, Jin L, Zhang Y, Huang Y, Zhang F, Valdmanis PN, and Kay MA. The loop position of shRNAs and pre-miRNAs is critical for the accuracy of dicer processing in vivo. *Cell*. 2012;151(4):900-11.
18. Huo W, Zhao G, Yin J, Ouyang X, Wang Y, Yang C, Wang B, Dong P, Wang Z, Watari H, et al. Lentiviral CRISPR/Cas9 vector mediated miR-21 gene editing inhibits the epithelial to mesenchymal transition in ovarian cancer cells. *J Cancer*. 2017;8(1):57-64.
19. Dalton HJ, Armaiz-Pena GN, Gonzalez-Villasana V, Lopez-Berestein G, Bar-Eli M, and Sood AK. Monocyte subpopulations in angiogenesis. *Cancer Res*. 2014;74(5):1287-93.

Supplemental Figure Legends

Figure S1. Analysis Tumor immune infiltrate in *miR-21*$^{-/-}$ mice or WT mice adoptively transferred with *miR-21*$^{-/-}$ BM. (**A, B, D**) Flow cytometry analysis of immune cell infiltrate in s.c. LLC tumors of *WT* or *miR-21*$^{-/-}$ mice (n=4 out 5 randomly selected, representative experiments out of three with similar results). (**A**) average % of MDSCs. Left (Total): Gated on $CD45^+CD11b^+Gr1^+$. Middle (monocytic): $CD45^+CD11b^+LY6C^{high}LY6G^-$. Right (granulocytic): $CD45^+CD11b^+LY6G^{high}$ $LY6C^-$ cells. (n=4). (**B**) Left: Average % of $CD4^+$TILs. Right: $CD8^+$TILs. (n=4). (**C, E**) Flow cytometry analysis of immune cell infiltrate in s.c. LLC tumors of *WT* mice transplanted with *WT* or *miR-21*$^{-/-}$ BM cells (n=5 out of 6 randomly selected, representative experiments out of two with similar results). (**C**) Left: Average % of $CD4^+$TILs and $CD8^+$TILs. (**D**) Flow cytometry analysis of IFNG expression after PMA/ION activation for 4 hrs in $CD4^+$TILs (Left), and $CD8^+$TILs (Right). (**E**) Left: Average % TAMs expressing CCR2. Middle: Representative histograms of CCR2 surface expression in TAMs. Right: average of geometric mean fluorescent intensity of CCR2 surface expression. Results are mean ± SEM. $^*P < 0.05$. Mann-Whitney U test.

Figure S2. miR-21 qISH assay validation and miR-21 expression in BMDMs and TAMs. (**A**) miR-21 ISH (green) on heart and spleen tissue of *miR-21*$^{-/-}$ or *WT* mice merged with DAPI (blue). qRT-PCR analysis of mature miR-21 levels (normalized to U6 RNA) in the indicated cells in culture (**B**) or in different cells isolated from s.c. eGFP-LLC tumors of WT mice (**C**). (n=3). Data correspond to the mean ± SEM. $^*P < 0.05$. Kruskal-Wallis test . Representative experiment out of two with similar results.

Figure S3. Gene expression analysis of *miR-21*$^{-/-}$ TAMs. (**A**) Gene expression patterns in *WT* and *miR-21*$^{-/-}$ TAMs in principal component analysis. (**B-D**) Heatmaps with the list of genes involved in: regulating the formation of endothelial tube (**B**), function of macrophages (**C**), and activation of lymphocytes (**D**), that are differentially expressed in TAMs of *WT* and *miR-21*$^{-/-}$ LLC TAMs. **E)** Normalized mRNA counts (log2-transformed gene expression values, RPKMs) of the indicated genes of *WT* or *miR-21*$^{-/-}$ TAMs of LLC tumors from *WT* or *miR-21*$^{-/-}$ mice, respectively. (**F**) qRT-PCR analysis of relative mRNA levels of *Il12*, *Tnf*, and *Cxcl10*

(normalized to 18S rRNA) in *WT* or *miR-21*$^{-/-}$ BMDMs after 6hrs of LPS activation (100ng/ml) (n=3). **G**) % CD8^{+} T-cells expressing GZMB in Peyer's patches of healthy *WT* or *miR-21*$^{-/-}$ mice (n=3). (**H**) % CD8^{+} splenocytes of *WT* and *miR-21*$^{-/-}$ mice expressing GZMB, 3 and 6 days post-activation with anti-CD3-antibody bound-plates (n=4). (F-H) Results are mean ± SEM. $^{*}P <$ 0.05. (**F-H**) Representative experiment out of two with similar results. (**F**) Kruskal-Wallis test. (**G**) Mann-Whitney U test. (**H**) two-way ANOVA with Bonferroni correction.

Figure S4. Expression of miR-21 in TMAs or DCs, analysis of cell death in TECs of tumors from *miR-21*$^{fl/fl}$ and *LysMCre;miR-21*$^{f/f}$ and tumor growth of B16 melanoma skin tumors in *miR-21*$^{fl/fl}$ and *LysMCre;miR-21*$^{f/f}$ (**A**) Left: Representative images of immunofluorescence co-staining of CD31 and TUNEL in LLC tumor cross sections of *miR-21*$^{fl/fl}$ and *LysMCre;miR-21*$^{f/f}$ mice. Right: average % of the quantification of double positive structures (n=7). (**B** & **C**) Tumor analysis of *miR-21*$^{fl/fl}$ *and LysMCre;miR-21*$^{f/f}$ mice with subcutaneous (s.c.) injection of B16 melanoma in the dorsal flank (n=5). Tumor volume (**B**) and final tumor weight (**C**). (**D**) qRT-PCR analysis of mature miR-21 levels (normalized to U6 RNA) in TAMs or DCs sorted from s.c. LLC tumors of *WT* or *LysMCre;miR-21*$^{f/f}$ mice (n=3). Scale bars, 70μm. Results are mean ± SEM. $^{*}P < 0.05$. (**A** & **C**) Mann-Whitney U test. (**B**) Two-way ANOVA (time and genotype) with Bonferroni correction **(D)** Kruskal-Wallis test., $^{\#}P < 0.05$ individual comparisons. Representative experiment out of two with similar results.

Figure S5. ClusterMap analysis of tumor CD45^{+} cells from *miR-21*$^{fl/fl}$ and *LysMCre;miR-21*$^{f/f}$ datasets. (**A**) All events were positive for Ptcpr (CD45^{+}) purple. Based on the known markers CD8^{+}T cells, CD4^{+} T cells, natural killer (NK) cells, granulocytic MDSCs, monocytic MDSCs, dendritic cells (DCs) and macrophages/monocytes were assigned (purple= positive; Gray:negative). Group 1: CD8^{+}T cells(*Cd3d*$^{+}$*, Cd3e*$^{+}$*, Cd3g*$^{+}$*, Cd8a*$^{+}$*,Cd8b1*$^{+}$*, Nkg7*$^{+}$), Group 2: CD4^{+}T cells (*Cd3d*$^{+}$*, Cd3e*$^{+}$*, Cd3g*$^{+}$*, Cd4*$^{+}$*, Nkg7*$^{+}$), Group 3: NK cells (*Nkg7*$^{+}$*,Cd38*$^{+}$*, Itgax*$^{+}$*, Itgam*$^{-}$*, Cd68*$^{-}$), Group4: gMDSCs (*S100Aa*$^{+}$*, Cd33*$^{+}$*, Ly6g*$^{+}$*, Adgre1*$^{-}$*, Ly6c*$^{-}$), Group 5: mMDSCs (*S100Aa*$^{+}$*, Cd33*$^{+}$*, Ly6g*$^{-}$*, Adgre1*$^{-}$*, Ly6c*+), Group 6: DCs (*Ly75*$^{+}$*, Ccr7*$^{+}$*, Fscn1*$^{+}$*, csf1r*$^{-}$*,Cd68, Ccr2*$^{-}$), mixture of macrophages/monocytes (*Adgre*$^{+}$*, Cd68*$^{+}$*, Csf1r*$^{+}$*,Ly6g*$^{-}$*, Ly6c2*$^{+}$*, Lyz2, Nkg7*$^{-}$*, Cd3*$^{-}$*, Cd4*$^{-}$), Group # B cells, that was identified as a unique entry positive (*Cd19*$^{+}$*,Nkg7*$^{-}$*,Adgre*$^{-}$ *, Cd68*$^{-}$). (**B**) ClusterMap analysis of Mixture of Macrophages/Monocytes, based on known

markers for TAMs (19): Group a: Pro-angiogenic -M2 "like" (*Vegfa*$^+$, *Arg1*$^+$, *Ccr2*$^+$, *Cxcl2*$^+$). Group b: Pro-inflammatory (*Cxcl10*$^+$, *Cxcl9*$^+$, *Isg15*$^+$). The rest of the populations remained as Unassigned given the overlap in the expression of different makers. *Cxc3r1, Chil3, Cd63* reported pro-angiogenic markers overlapped with pro-inflammatory population and reported inflammatory markers *Ccl4, Tlr4, Tlr2, Cd86, Cd40* overlapped with pro-angiogenic populations.

Figure S6. Analysis of cell incorporation of AF546-pHLIP or pHLIP-anti-miR-21-TAMRA by different cells. (**A-C**) AF546 pHLIP Var3 uptake measured by flow cytometry in the indicated cells isolated from s.c. eGFP-LLC tumors of WT mice. AF546-pHLIP was injected i.v. 12 days post tumor cell injection (n=3). Representative cell plots (**A**) and histograms (**B**), respectively. (**C**) Quantification of AF546 cell incorporation. Data are geometric mean intensity of fluorescence after subtracting the fluorescence of cells that were obtained from control injected mice. (**D**) Representative overlayed histograms of flow cytometry analysis of the uptake of pHLIP-anti-miR-21-TAMRA by TAMs, DCs and other leukocytes (no TAMs or DCs) of s.c. LLC tumors of *WT* mice (n=6) as in Figure 7. (**E**) Quantification of TAMRA cell incorporation. Data are geometric mean intensity of fluorescence after subtracting the fluorescence of cells that were obtained from control injected mice. (**F**) Representative images of immunofluorescence staining of CD68, CD31 and TAMRA detection in cross sections of s.c. LLC tumors of WT mice treated with pHLIP-anti-miR-21-TAMRA. (G) qRT-PCR analysis of mature miR-21 levels (normalized to U6 RNA) or *Il12*, *Tnf*, and *Cxcl10* mRNA levels (normalized to 18S RNA) in sorted TAMs (CD45$^+$CD11b$^+$MHCII$^+$F4/80$^+$) or DCs (CD45$^+$CD11b$^+$CD11c$^+$MHCII$^+$F4/80$^-$ from s.c. LLC tumors of *WT* mice treated as indicated (n=4). Results are mean ± SEM. $^*P < 0.05$. (**C**, **E, G**) Kruskal-Wallis test. Scale bars, 150μm.

Figure S7. Generation and characterization of *miR-21*$^{-/-}$ LLCs. (**A**) Top: pre-miR-21 sequence from mirbase.com. Bottom: seed and PAM sequence of miR-21 and gRNA used to induce a dsDNA break. (**B**) q-RT-PCR analysis of, Left: pri-miR-21, Middle: pre-miR-21, Right: mature miR-21 levels in LLCs or *miR-21*$^{-/-}$ LLCs (normalized to 18 S rRNA). (**C**) qRT-PCR analysis of *pten* mRNA levels in LLCs and *miR-21*$^{-/-}$ LLCs transfected with miR-21 mimics or inhibitors, or their respective controls (normalized to 18 S rRNA). Analysis was performed 48

hrs post transfection. (**D**) Total number of cells 72 hrs post-transfection of LLCs or *miR-21*$^{-/-}$ LLCs with, Left: miR-21 mimic or control mimic, as indicated, Right: miR-21 inhibitor or control inhibitor, as indicated. Results are representative of one experiment out of three with similar results and are mean of triplicate conditions ± SD.

Figure S8. Analysis of tumor growth in pHLIP-anti-miR-21 treated mice. (**A**) Bioluminescence imaging of lung tumors generated by injecting LL/2Red Luc cells into the lungs of WT mice treated with 1mg/kg pHLIP-anti-miR-21 or pHLIP-anti-miR-21-mismatch control on indicated days (n=7 and 10). (**B**) Final tumor weight (for tumor weight remaining healthy lung tissue was removed). (**C**) Detection of Luciferase and TAMRA fluorescence in lung tumors and selected organs 72h after last i.v. injection of pHLIP-PNA-TAMRA. Results are mean ± SEM. $^{*}P < 0.05$. (**A**) two-way ANOVA (time and genotype) with Bonferroni correction, $^{\#}P < 0.05$ individual comparisons. (**B**) Mann-Whitney U test. Representative experiment out of two with similar results.

Figure S1

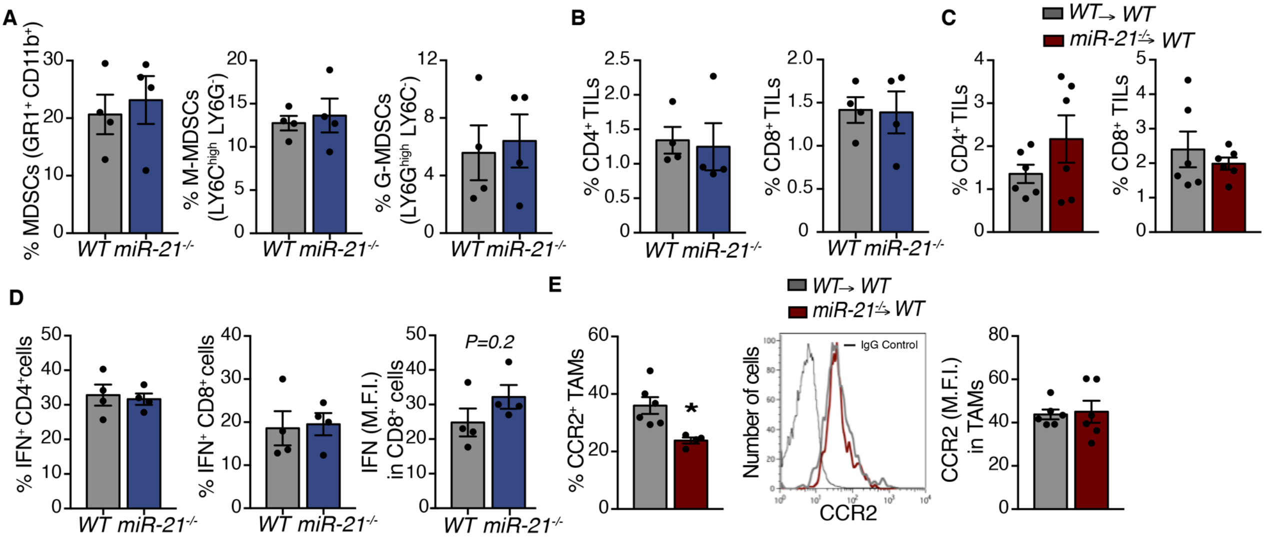

A
% MDSCs (GR1+ CD11b+)
% M-MDSCs (LY6Chigh LY6G-)
% G-MDSCs (LY6Ghigh LY6C-)
WT miR-21-/-
B
% CD4+ TILs
% CD8+ TILs
C
WT→ WT
miR-21-/-→ WT
D
% IFN+ CD4+cells
% IFN+ CD8+ cells
IFN (M.F.I.) in CD8+ cells
P=0.2
E
% CCR2+ TAMs
Number of cells
IgG Control
CCR2
CCR2 (M.F.I.) in TAMs

Figure S2

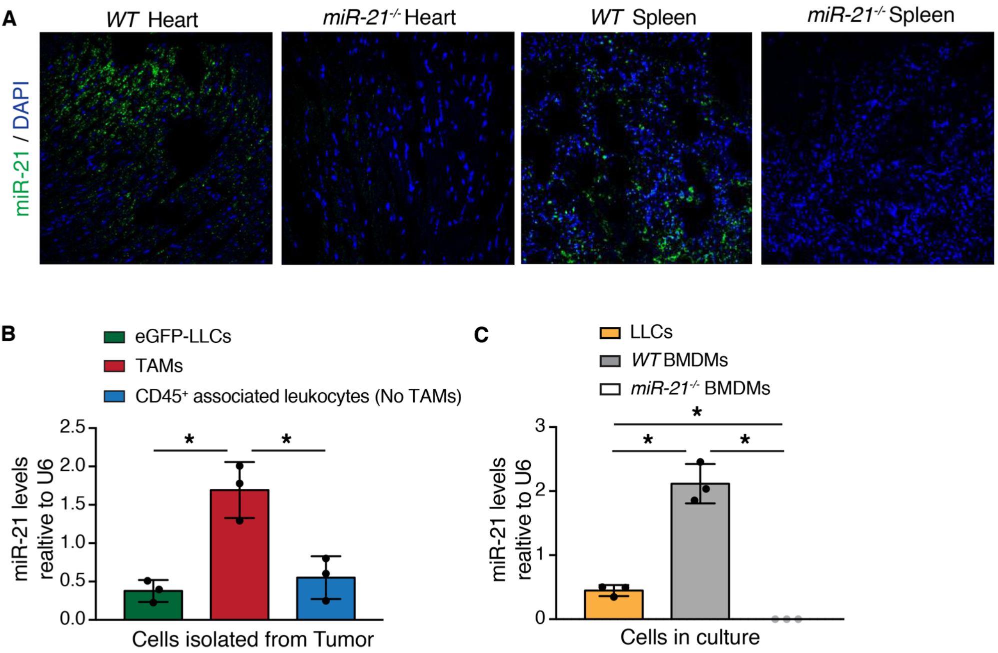
A
WT Heart
miR-21-/- Heart
WT Spleen
miR-21-/- Spleen
miR-21 / DAPI
B
eGFP-LLCs
TAMs
CD45+ associated leukocytes (No TAMs)
miR-21 levels realtive to U6
2.5
2.0
1.5
1.0
0.5
0.0
*
*
Cells isolated from Tumor
C
LLCs
WT BMDMs
miR-21-/- BMDMs
miR-21 levels realtive to U6
3
2
1
0
*
*
*
Cells in culture

Figure S3

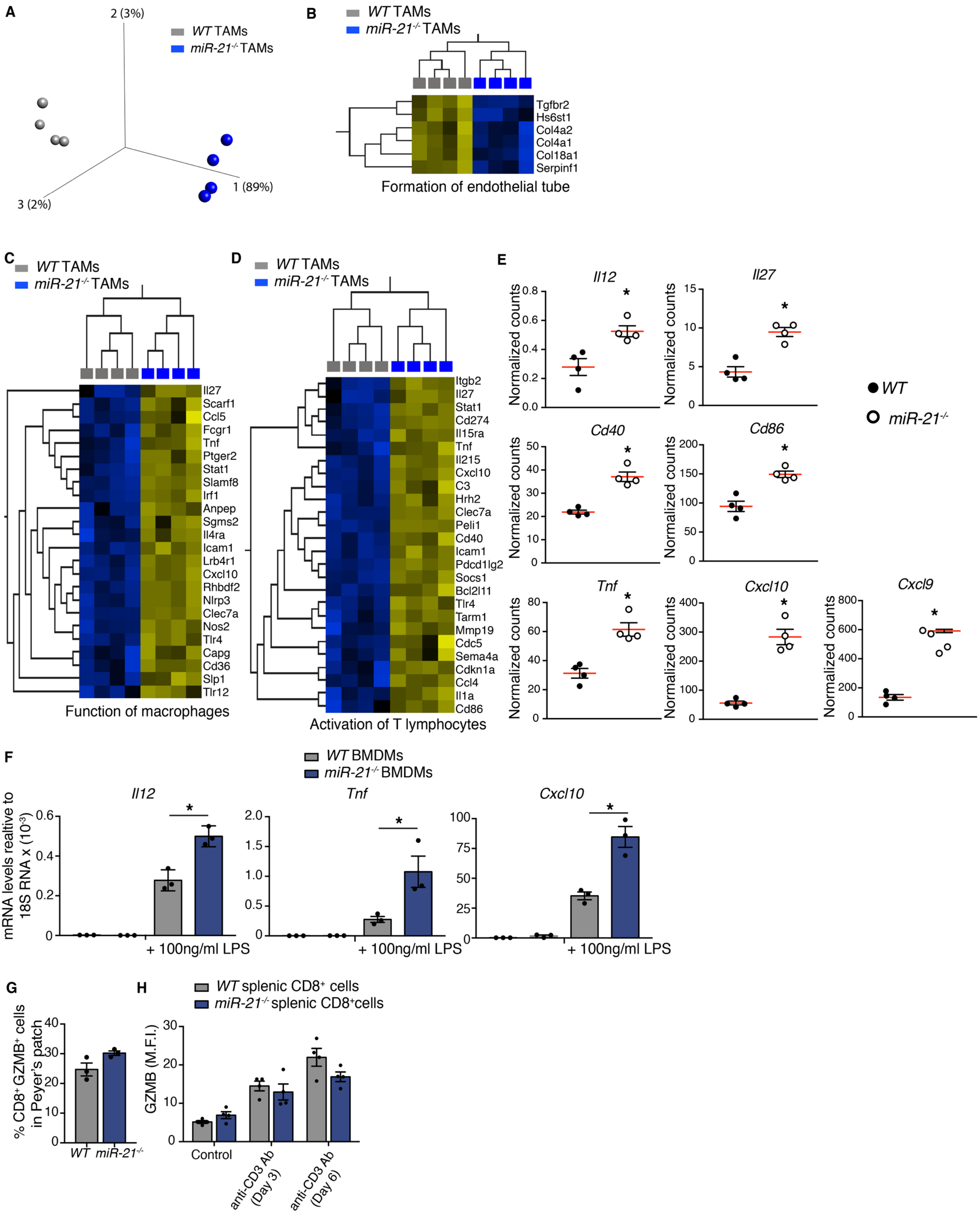

A
2 (3%)
WT TAMs
miR-21-/- TAMs
1 (89%)
3 (2%)
B
WT TAMs
miR-21-/- TAMs
Tgfbr2
Hs6st1
Col4a2
Col4a1
Col18a1
Serpinf1
Formation of endothelial tube
C
WT TAMs
miR-21-/- TAMs
Il27
Scarf1
Ccl5
Fcgr1
Tnf
Ptger2
Stat1
Slamf8
Irf1
Anpep
Sgms2
Il4ra
Icam1
Lrb4r1
Cxcl10
Rhbdf2
Nlrp3
Clec7a
Nos2
Tlr4
Capg
Cd36
Slp1
Tlr12
Function of macrophages
D
WT TAMs
miR-21-/- TAMs
Itgb2
Il27
Stat1
Cd274
Il15ra
Tnf
Il215
Cxcl10
C3
Hrh2
Clec7a
Peli1
Cd40
Icam1
Pdcd1lg2
Socs1
Bcl2l11
Tlr4
Tarm1
Mmp19
Cdc5
Sema4a
Cdkn1a
Ccl4
Il1a
Cd86
Activation of T lymphocytes
E
Il12
Il27
Cd40
Cd86
Tnf
Cxcl10
Cxcl9
Normalized counts
WT
miR-21-/-
F
WT BMDMs
miR-21-/- BMDMs
Il12
Tnf
Cxcl10
mRNA levels realtive to 18S RNA x (10-3)
+ 100ng/ml LPS
G
% CD8+ GZMB+ cells in Peyer's patch
WT
miR-21-/-
H
WT splenic CD8+ cells
miR-21-/- splenic CD8+cells
GZMB (M.F.I.)
Control
anti-CD3 Ab (Day 3)
anti-CD3 Ab (Day 6)

Figure S4

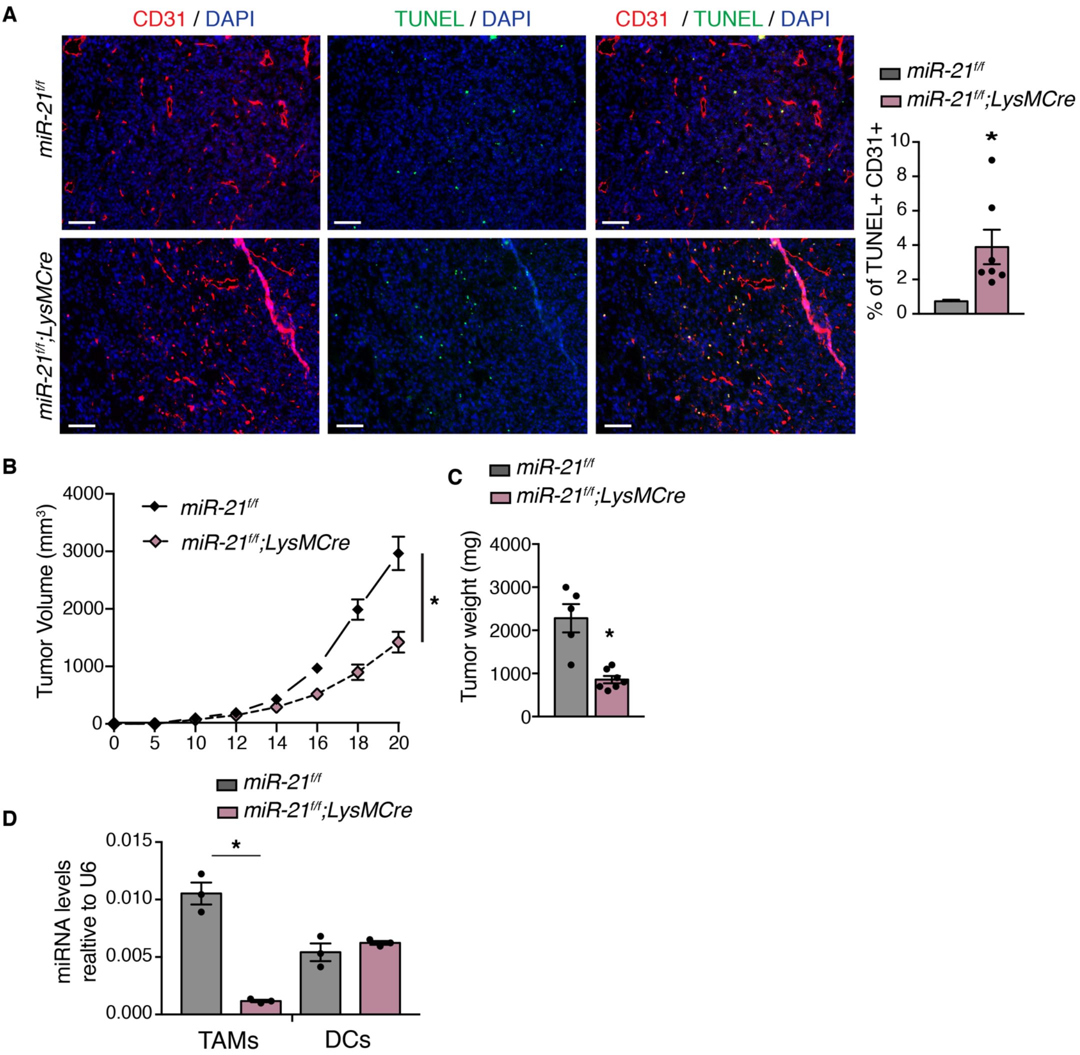
A
CD31 / DAPI
TUNEL / DAPI
CD31 / TUNEL / DAPI
miR-21f/f
miR-21f/f;LysMCre
% of TUNEL+ CD31+
B
Tumor Volume (mm3)
C
Tumor weight (mg)
D
miRNA levels realtive to U6
TAMs
DCs

Figure S5

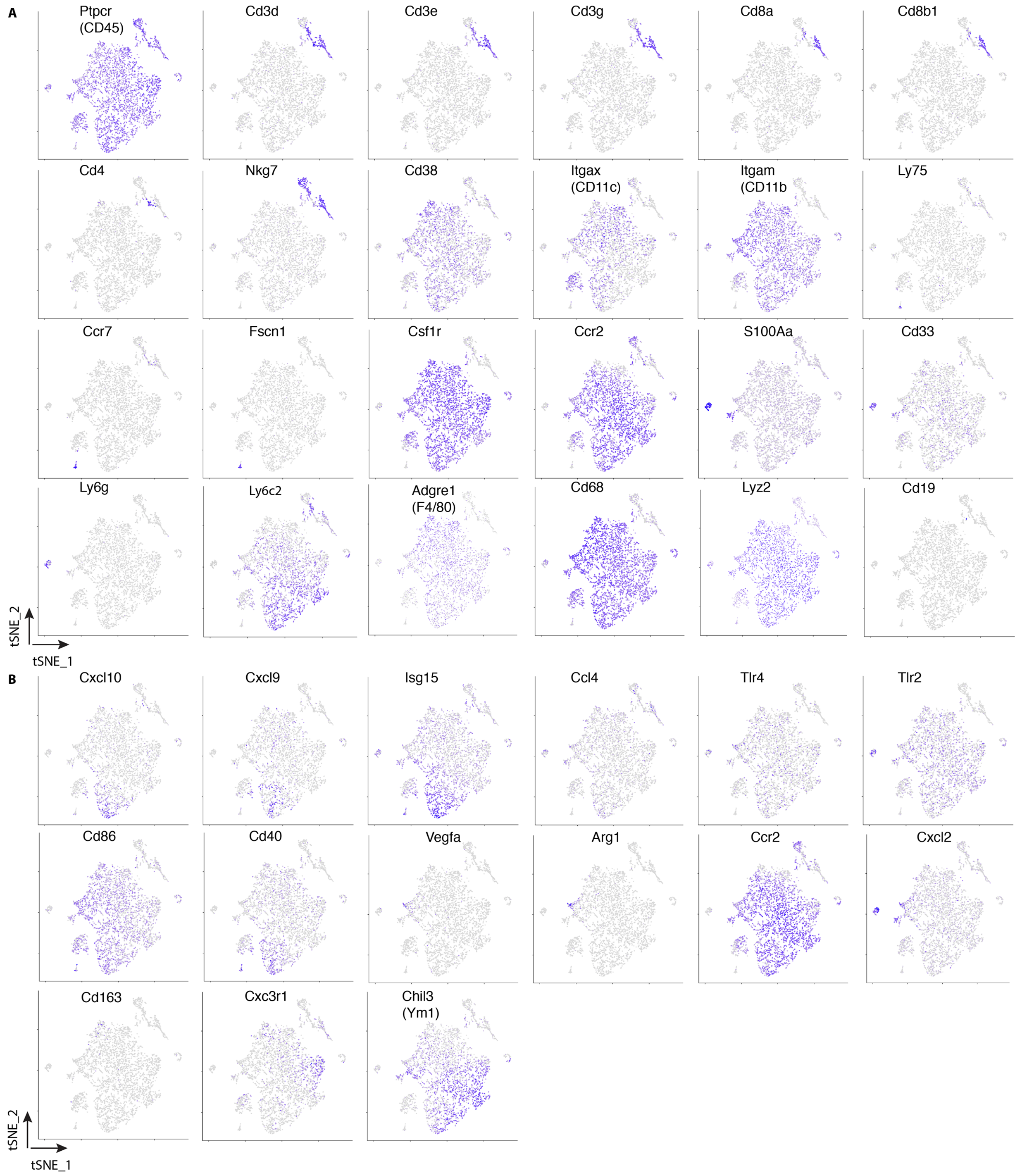
A
Ptpcr
(CD45)
Cd3d
Cd3e
Cd3g
Cd8a
Cd8b1
Cd4
Nkg7
Cd38
Itgax
(CD11c)
Itgam
(CD11b
Ly75
Ccr7
Fscn1
Csf1r
Ccr2
S100Aa
Cd33
Ly6g
Ly6c2
Adgre1
(F4/80)
Cd68
Lyz2
Cd19
tSNE_2
tSNE_1
B
Cxcl10
Cxcl9
Isg15
Ccl4
Tlr4
Tlr2
Cd86
Cd40
Vegfa
Arg1
Ccr2
Cxcl2
Cd163
Cxc3r1
Chil3
(Ym1)
tSNE_2
tSNE_1

Figure S6

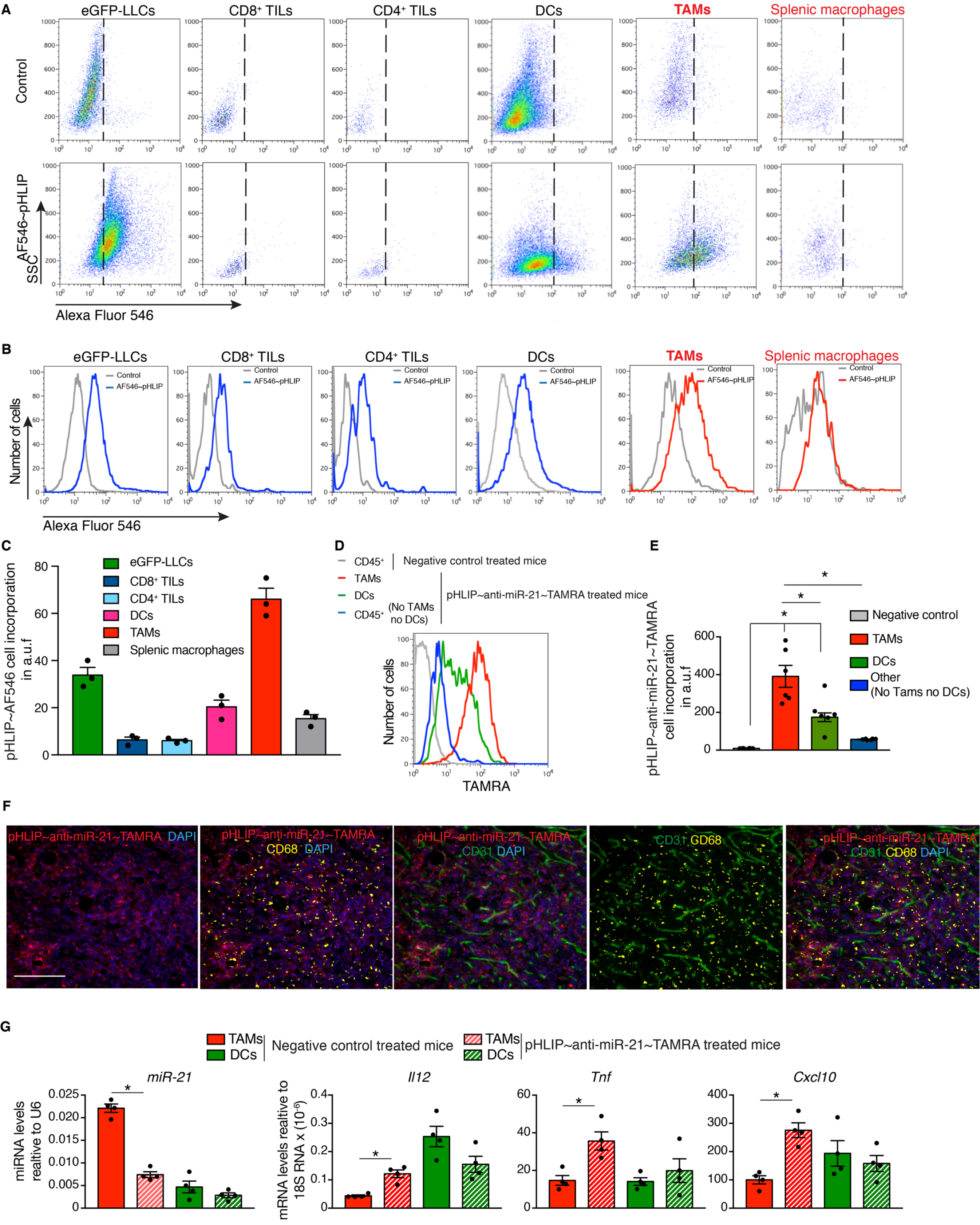
A
eGFP-LLCs
CD8⁺ TILs
CD4⁺ TILs
DCs
TAMs
Splenic macrophages
Control
AF546~pHLIP
SSC
Alexa Fluor 546
B
Control
AF546~pHLIP
Number of cells
Alexa Fluor 546
C
eGFP-LLCs
CD8⁺ TILs
CD4⁺ TILs
DCs
TAMs
Splenic macrophages
pHLIP~AF546 cell incorporation in a.u.f
D
CD45⁺
TAMs
DCs
CD45⁺ (No TAMs no DCs)
Negative control treated mice
pHLIP~anti-miR-21~TAMRA treated mice
Number of cells
TAMRA
E
Negative control
TAMs
DCs
Other (No Tams no DCs)
pHLIP~anti-miR-21~TAMRA cell incorporation in a.u.f
F
pHLIP~anti-miR-21~TAMRA DAPI
pHLIP~anti-miR-21~TAMRA CD68 DAPI
pHLIP~anti-miR-21~TAMRA CD31 DAPI
CD31 CD68
pHLIP~anti-miR-21~TAMRA CD31 CD68 DAPI
G
TAMs
DCs
Negative control treated mice
pHLIP~anti-miR-21~TAMRA treated mice
miR-21
Il12
Tnf
Cxcl10
miRNA levels realtive to U6
mRNA levels realtive to 18S RNA x (10⁻⁶)

Figure S7

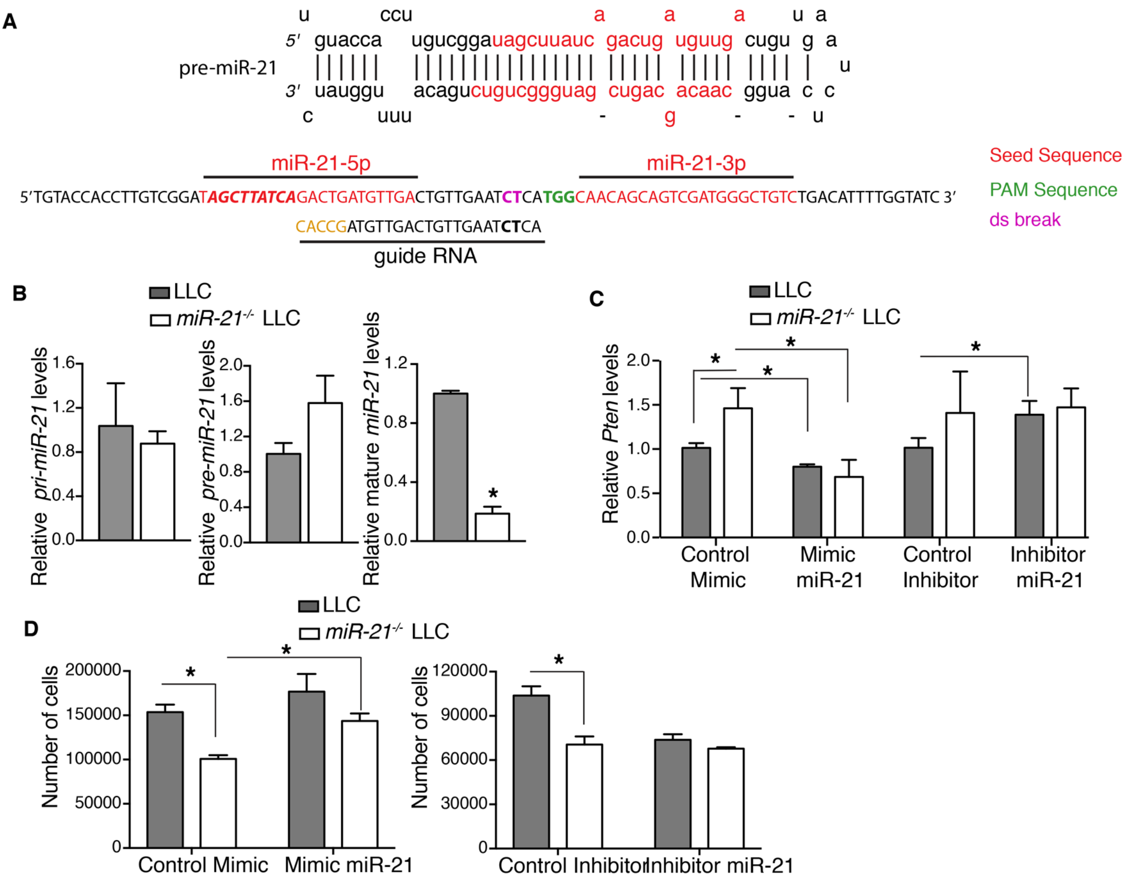

A
pre-miR-21
u ccu a a a u a
5′ guacca ugucggauagcuuauc gacug uguug cugu g a
u
3′ uauggu acagucugucggguag cugac acaac ggua c c
c uuu - g - - u
miR-21-5p
miR-21-3p
5′TGTACCACCTTGTCGGATAGCTTATCAGACTGATGTTGACTGTTGAATCTCATGGCAACAGCAGTCGATGGGCTGTCTGACATTTTGGTATC 3′
CACCGATGTTGACTGTTGAATCTCA
guide RNA
Seed Sequence
PAM Sequence
ds break
B
LLC
miR-21-/- LLC
Relative pri-miR-21 levels
Relative pre-miR-21 levels
Relative mature miR-21 levels
*
C
LLC
miR-21-/- LLC
Relative Pten levels
*
*
*
*
Control Mimic
Mimic miR-21
Control Inhibitor
Inhibitor miR-21
D
LLC
miR-21-/- LLC
Number of cells
*
*
Control Mimic
Mimic miR-21
*
Control Inhibitor
Inhibitor miR-21

Figure S8

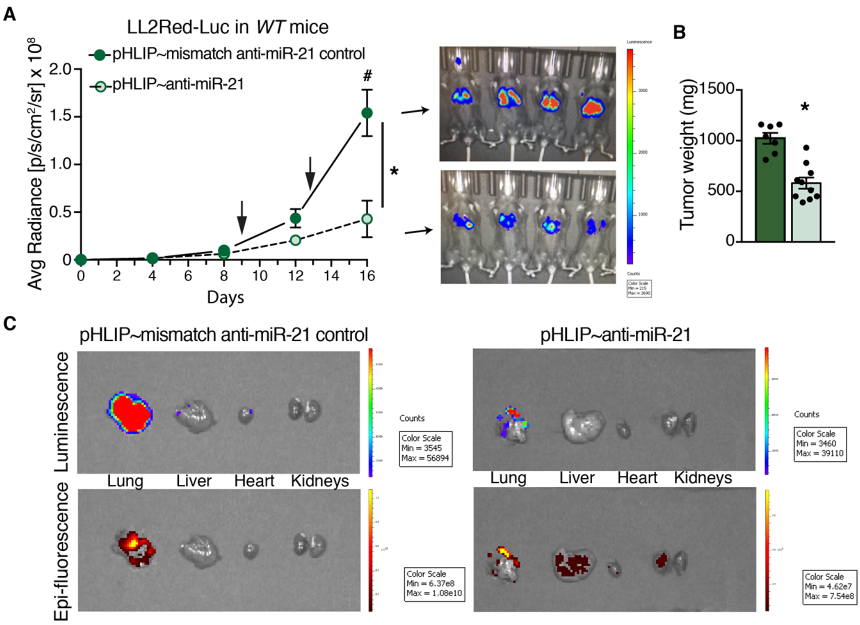
A
LL2Red-Luc in WT mice
pHLIP~mismatch anti-miR-21 control
pHLIP~anti-miR-21
Avg Radiance [p/s/cm²/sr] x 10⁸
2.0
1.5
1.0
0.5
0
0
2
4
6
8
10
12
14
16
Days
#
*
Counts
B
Tumor weight (mg)
1500
1000
500
0
*
C
pHLIP~mismatch anti-miR-21 control
pHLIP~anti-miR-21
Luminescence
Epi-fluorescence
Lung
Liver
Heart
Kidneys
Counts
Color Scale
Min = 3545
Max = 56894
Counts
Color Scale
Min = 3460
Max = 39110
Color Scale
Min = 6.37e8
Max = 1.08e10
Color Scale
Min = 4.62e7
Max = 7.54e8

Supplemental Tables

Table S1. Demographics and Clinicopathological characteristics of NSCLC Yale cohort YTMA79

Characteristic	N (%)
**Total**	178
**Gender**	
Male	85 (40.0)
Female	93 (60.0)
**Age**	
< 70 yo	116(56.4)
>= 70 yo	62 (43.6)
**Stage**	
I-II	129 (77.8)
III-IV	48 (2.0)
Unknown	1
**Histology**	
ADC	122 (60.0)
SCC	27 (26.4)
Other	29 (13.6)
**Smoking History**	
Non smoker	14 (12.1)
Current/Former	161(82.9)
Unknown	3

Table S2. **Demographics and Clinicopathological characteristics of NSCLC Yale cohort YTMA140**

Characteristic	N (%)
**Total**	344
**Gender**	
Male	302 (40.0)
Female	42 (60.0)
**Age**	
< 70 yo	265 (56.4)
>= 70 yo	79 (43.6)
**Stage**	
I-II	197 (77.8)
III-IV	140 (2.0)
Unknown	7
**Histology**	
ADC	141 (60.0)
SCC	162 (26.4)
Other	41 (13.6)
**Smoking History**	
Non smoker	29 (12.1)
Current/Former	279 (82.9)
Unknown	36

Table S3. **Demographics and Clinicopathological characteristics of NSCLC Yale cohort YTMA250**

Characteristic	N (%)
**Total**	263
**Gender**	
Male	116 (40.0)
Female	147 (60.0)
**Age**	153 (56.4)
< 70 yo	110 (43.6)
>= 70 yo	
**Stage**	
I-II	208 (77.8)
III-IV	49 (2.0)
Unknown	6
**Histology**	
ADC	159 (60.0)
SCC	68 (26.4)
Other	36 (13.6)
**Smoking History**	
Non smoker	42 (12.1)
Current/Former	206 (82.9)
Unknown	15
